# Neutral sphingomyelinase 2 controls exosome secretion by counteracting V-ATPase-mediated endosome acidification

**DOI:** 10.1242/jcs.259324

**Published:** 2022-02-28

**Authors:** Dolma Choezom, Julia Christina Gross

**Affiliations:** 1 Developmental Biochemistry, University Medical Center Goettingen, 37077 Goettingen, Germany; 2 Hematology and Oncology, University Medical Center Goettingen, 37075, Goettingen, Germany; 3 Biochemistry Department, Health and Medical University, 14471 Potsdam, Germany

**Keywords:** Small extracellular vesicles, Endosomal maturation, Secretory multivesicular bodies, Intraluminal vesicles, Endosomal cargo sorting

## Abstract

During endosome maturation, neutral sphingomyelinase 2 (nSMase2, encoded by *SMPD3*) is involved in budding of intraluminal vesicles (ILVs) into late endosomes or multivesicular bodies (MVBs). Fusion of these with the plasma membrane results in secretion of exosomes or small extracellular vesicles (sEVs). Here, we report that nSMase2 activity controls sEV secretion through modulation of vacuolar H^+^-ATPase (V-ATPase) activity. Specifically, we show that nSMase2 inhibition induces V-ATPase complex assembly that drives MVB lumen acidification and consequently reduces sEV secretion. Conversely, we further demonstrate that stimulating nSMase2 activity with the inflammatory cytokine TNFα (also known as TNF) decreases acidification and increases sEV secretion. Thus, we find that nSMase2 activity affects MVB membrane lipid composition to counteract V-ATPase-mediated endosome acidification, thereby shifting MVB fate towards sEV secretion.

This article has an associated First Person interview with the first author of the paper.

## Abstract (precis)

**Summary:** Changing neutral sphingomyelinase 2 activity regulates small extracellular vesicle secretion through modulation of V-ATPase activity.

## INTRODUCTION

Exosomes are small extracellular vesicles (sEVs) of endosomal origin that are released into the extracellular space and contain bioactive signaling molecules (Ciardiello et al., 2016). Initially considered cellular waste, recent studies have proven that sEVs play key roles in cell–cell communication, for example during erythrocyte maturation, antigen presentation and progression of various diseases including Alzheimer's disease and cancer (Danzer et al., 2012; Bebelman et al., 2018). Cancer cells highly upregulate exosome secretion, and this significantly contributes to tumor progression by mediating different processes, including proliferation, metastasis and organ tropism (Kanada et al., 2016). Therefore, it is crucial to understand the molecular mechanisms that underlie exosome biogenesis and its regulation.

Exosomes are generated as intraluminal vesicles (ILVs) during endosome maturation into late endosomes or multivesicular bodies (MVBs). ILVs are secreted out as exosomes into the extracellular space when MVBs fuse with the plasma membrane. As early endosomes mature into MVBs, cargoes are sorted on the endosomal membrane, which then buds inward to form ILVs (van Niel et al., 2018). Different molecular machineries and pathways are involved in ILV biogenesis. As one route of biogenesis, the syndecan–syntenin-1–Alix (also known as PDCD6IP) axis engages with the endosomal sorting complex required for transport (ESCRT) machinery to generate ILVs for exosome secretion (Baietti et al., 2012). Interestingly, the ESCRT-machinery also generates ILVs in MVBs that are targeted for lysosomal degradation. For example, the ubiquitylated epidermal growth factor receptors (EGFRs) that are internalized into ILVs in an ESCRT-dependent manner are targeted for lysosomal degradation (Roxrud et al., 2008; Raiborg and Stenmark, 2009).

In addition, ILVs can be generated by ESCRT-independent pathways that involve neutral sphingomyelinase 2 (nSMase2, encoded by *SMPD3*). nSMase2 hydrolyzes sphingomyelin into ceramide and phosphorylcholine (Hofmann et al., 2000). In a mouse oligodendroglial cell line, it has been shown that ceramide produced by nSMase2 is required for sorting proteolipid protein (PLP) into ILVs destined for secretion (Trajkovic et al., 2008). Complementary *in vitro* results have shown the formation of intravesicular membranes upon ceramide generation in giant unilamellar vesicles (GUVs). Accordingly, it has been argued that ceramide generated at the limiting membrane of MVBs drives spontaneous negative membrane curvature and budding, leading to the formation of ILVs. Additionally, a recent study has shown that the ceramide metabolite sphingosine-1-phosphate activates its receptor on MVBs to segregate cargoes into ILVs for exosome secretion (Kajimoto et al., 2013). Whether ILV formation by nSMase2 is independent of ESCRT proteins or whether they cooperate at the same MVBs for ILV formation and cargo loading is so far not well understood.

Similarly, it is unclear what discriminates and controls the fate of MVBs. MVBs can either be sorted for lysosomal degradation (degradative MVBs) or be transported towards the cell periphery for plasma membrane fusion (secretory MVBs) to release exosomes (van Niel et al., 2018). Regulatory factors that control the fate of MVB trafficking after ILV generation and their subsequent fusion with the plasma membrane remain largely unknown. Interestingly, numerous studies have established a connection between endosomal acidification and sEV secretion – decreasing endosomal acidification increases exosome secretion (Villarroya-Beltri et al., 2016; Edgar et al., 2016; Guo et al., 2017; Latifkar et al., 2019). The vacuolar H^+^-ATPase (V-ATPase) is a multi-subunit proton pump responsible for endosomal acidification (Forgac, 2007). Cancer cells downregulate sirtuin-1 (SIRT-1), which destabilizes the mRNA of ATP6V1A, a subunit of the complex (Latifkar et al., 2019). This impairs V-ATPase activity that acidifies the MVB lumen and instead favors the sorting of these MVBs for exosome release. Similarly, another study has shown that autophagy-related gene 5 (ATG5), a protein involved in the autophagy pathway, dissociates the ATP6V1E1 subunit from the V-ATPase complex to reduce its activity and thus promote exosome secretion (Guo et al., 2017). Overall, these studies indicate that acidification plays a significant role in determining MVB sorting fate, with less acidic MVBs more likely to be targeted for secretion. Despite these recent findings, the molecular mechanism involved in fine-tuning MVB lumen acidification to regulate their sorting decision is not well understood.

We discovered that nSMase2 activity regulates endolysosomal acidification by modulating V-ATPase activity and thereby controls sEV secretion. Specifically, we show that nSMase2 inhibition and *SMPD3* knockdown renders the V-ATPase complex assembled and active for further MVB lumen acidification, which consequently reduces sEV secretion. Conversely, we further demonstrate that stimulating nSMase2 activity with the inflammatory cytokine TNFα (also known as TNF) decreases acidification and in turn increases sEV secretion. We show that nSMase2 activity counteracts V-ATPase-mediated endosome acidification and thereby shifts MVB fate towards sEV secretion.

## RESULTS

### nSMase2 regulates sEV secretion in HeLa cells

**Fig. 1.**
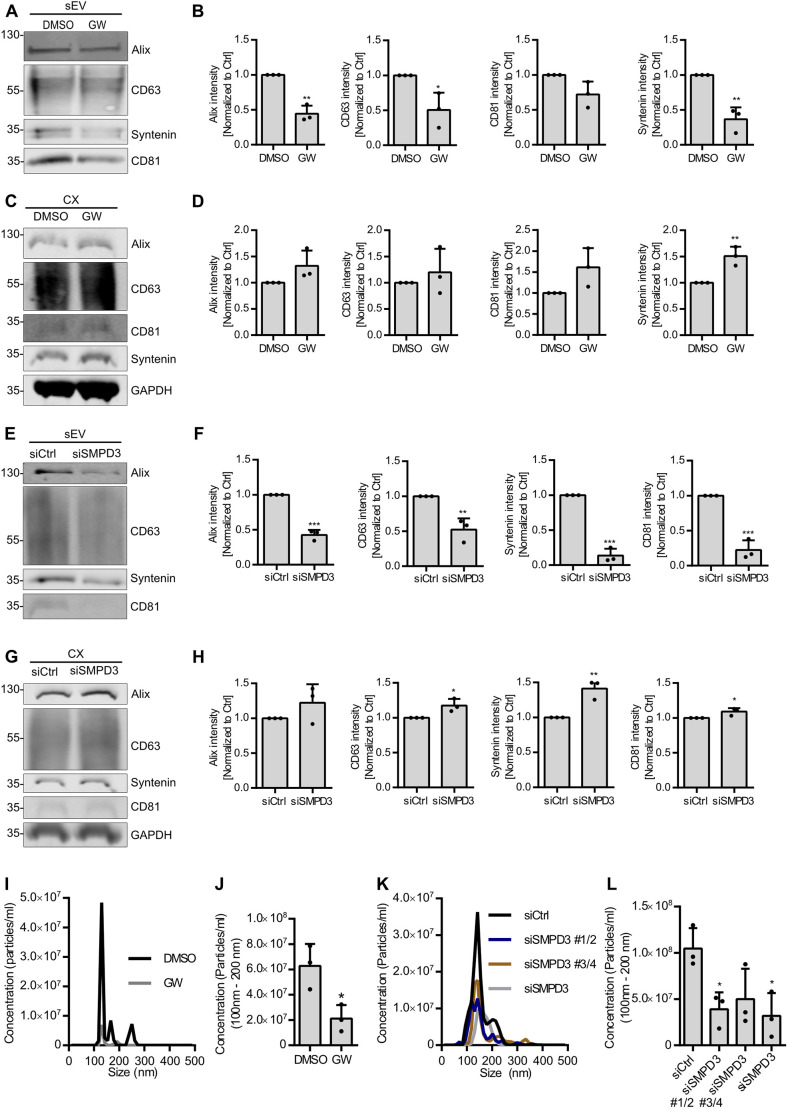
**nSMase2 regulates sEV secretion in HeLa cells.** (A) Western blot analysis of sEV fractions prepared from equal amounts of overnight DMSO- or GW4869 (GW)-treated HeLa cells. Samples were probed for the exosomal markers Alix, CD63, syntenin and CD81. (B) Signal intensity quantifications of Alix, CD63, syntenin and CD81 in sEV fractions from A, calculated by normalizing the signal to loading control GAPDH levels in the corresponding cell lysates in C before normalization to the respective control. (C) Western blot of the corresponding cell lysates (CX) from A, probed for loading control GAPDH in addition to the exosomal markers from A. (D) Quantifications of intracellular Alix, CD63, syntenin and CD81 signal intensity from C normalized to loading control GAPDH before normalization to the respective control. (E) Western blot analysis of sEV fractions prepared from equal amounts of control (siCtrl) and *SMPD3* KD (siSMPD3) HeLa cells, probed for the exosomal markers Alix, CD63, syntenin and CD81. (F) Signal intensity quantifications of Alix, CD63, syntenin and CD81 in sEV fractions from E, calculated by normalizing the signal to loading control GAPDH levels in the corresponding cell lysates in G before normalization to their respective control. (G) Western blot of the corresponding cell lysates (CX) from E, probed for loading control GAPDH in addition to the exosomal markers from E. (H) Quantifications of intracellular Alix, CD63, syntenin and CD81 signal intensity from G normalized to loading control GAPDH before normalization to the respective control. (I) Representative size distribution of sEVs prepared from DMSO- or GW4869-treated HeLa cells. (J) NTA quantification of sEV concentration from I. (K) Representative size distribution of sEVs isolated from control or *SMPD3* KD HeLa cells. *SMPD3* KD used either pairs of siRNAs (siSMPD3 #1/2 and siSMPD3 #3/4) or a pool of all four siRNAs (siSMPD3). (L) NTA quantification of sEV concentration from K. Data are presented as mean±s.d. of three biological replicates. ***P<0.0001; **P<0.001; *P<0.01 (unpaired, two-tailed Student's *t*-test in B,D,F,H,J; one-way ANOVA followed by Dunnett's comparison test in L). Molecular masses are indicated in kDa.

nSMase2 hydrolyzes sphingomyelin into ceramide and phosphorylcholine (Hofmann et al., 2000), a reaction that can be blocked by the inhibitor GW4869, which has been used in many studies to inhibit exosome secretion (Trajkovic et al., 2008; Menck et al., 2017; Li et al., 2016; Hu et al., 2019). However, several lines of evidence indicate that even after nSMase2 inhibition, exosome cargo loading, and thereby ILV generation, is not completely blocked (Gross et al., 2012). nSMase2 inhibition with GW4869 furthermore seems to affect large EV subpopulations and alter exosome composition, and even fails to reduce EV secretion in other cells (Menck et al., 2017; Leidal et al., 2020; Panigrahi et al., 2018). This prompted us to further dissect the role of nSMase2 in the endosomal pathway. As a first step, we analyzed sEV secretion upon nSMase2 inhibition with GW4869. Conditioned medium from equal numbers of HeLa cells treated with GW4869 or DMSO were subjected to serial ultracentrifugation to recover sEVs at 100,000 ***g*** (Fig. S1A). Following the guidelines recommended by the International Society for Extracellular Vesicles (ISEV; Théry et al., 2018), we hereafter refer to the isolated vesicles as sEVs rather than exosomes. Medium samples corresponding to equal amounts of cells were used to evaluate sEV secretion. Although HeLa cell viability remained unaffected (Fig. S1B), GW4869 treatment significantly reduced the secretion of Alix, syntenin-1 (encoded by *SDCBP* and referred to hereafter as syntenin) and CD63 in the sEV fraction (Fig. 1A,B). In line with their reduced secretion, GW4869 treatment slightly increased the intracellular levels of these markers (Fig. 1C,D). This indicates that due to their reduced secretion, the markers accumulated, possibly inside the endosomal pathway.

To exclude possible off-target effects of GW4869, we used siRNA against nSMase2 (targeting expression of *SMPD3*) to phenocopy the effects of the inhibitor. Knockdown (KD) of *SMDP3* was confirmed using qPCR (Fig. S1C) and did not affect the cell viability (Fig. S1D). *SMPD3* KD in HeLa cells significantly decreased the secretion of Alix, CD63, syntenin and CD81 in the sEV fraction (Fig. 1E,F) and significantly increased the corresponding intracellular levels of these exosomal markers (Fig. 1G,H). Moreover, nanotracking particle analysis (NTA) showed that most of the sEVs were between 100 nm and 200 nm in diameter (Fig. 1I,K) and confirmed their reduced secretion upon GW4869 treatment and *SMPD3* KD (Fig. 1J,L). Furthermore, KD using two separate sets of siRNAs against *SMPD3* or the combination thereof similarly reduced sEV secretion (Fig. 1L). Overall, these results further confirm the role of nSMase2 in sEV secretion in HeLa cells.

### *SMPD3* KD results in intracellular accumulation of MVBs

**Fig. 2.**
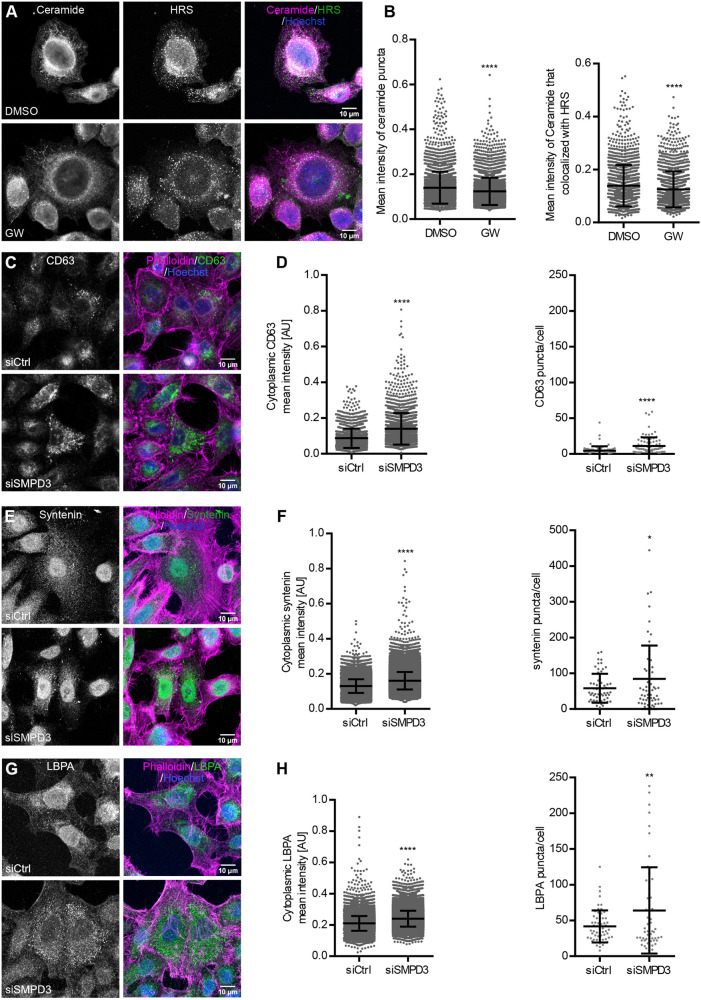
***SMPD3 KD* results in intracellular accumulation of MVBs.** (A) Confocal microscopy images of HeLa cells treated with DMSO or GW4869 (GW), stained for ceramide and HRS. Nuclei were stained with Hoechst. (B) Dot plots of mean fluorescence intensity of ceramide puncta (left) and mean fluorescence intensity of ceramide puncta that colocalized with HRS (right) from A. (C–H) Confocal microscopy images of control (siCtrl) and *SMPD3* KD (siSMPD3) HeLa cells stained for exosomal markers CD63 (C) and syntenin (E), and the MVB marker LBPA (G), as well as actin (phalloidin). Nuclei were stained with Hoechst. (D) Quantifications of cytoplasmic CD63 intensity (left) and CD63 puncta per cell (right) from C. (F) Quantifications of cytoplasmic syntenin intensity (left) and syntenin puncta per cell (right) from E. (H) Quantifications of cytoplasmic LBPA intensity (left) and LBPA puncta per cell (right) from (G). The data in B,D,F and H are presented as mean±s.d.; *n*>50 cells from two biological replicates for each staining. *P<0.01; **P<0.001; ****P<0.0001 (unpaired, two-tailed Student's *t*-test). AU, arbitrary units.

We reasoned that analyzing ceramide distribution in different organelles could shed light on the specific subcellular activity of nSMase2 and subsequent effects on MVB formation and exosomal biogenesis. As a readout for nSMase2 activity, we used an anti-ceramide antibody previously used for studies of sphingomyelin metabolism and ceramide signaling (Vielhaber et al., 2001; Parashuraman and D'Angelo, 2019; Yabu et al., 2015). Ceramide staining was analyzed in DMSO- and GW4869-treated HeLa cells using confocal microscopy. As expected, GW4869 treatment significantly reduced intracellular ceramide levels, suggesting reduced nSMase2 activity (Fig. 2A,B). As ceramide is also provided by *de novo* synthesis from the endoplasmic reticulum, where nSMase2 also localizes (Shamseddine et al., 2015), we co-stained ceramide with the endosomal ESCRT-0 component HRS (also known as HGS) to determine nSMase2 activity specifically at the endosomal membrane. GW4869 treatment significantly reduced the amount of ceramide colocalizing with HRS, confirming reduced nSMase2 activity at the endosomal level (Fig. 2A,B).

To further dissect the role of nSMase2 in sEV secretion, we next analyzed the intracellular distribution of selected exosomal markers in HeLa cells by confocal microscopy. Reduced staining of syntenin upon gene KD (*SDCBP* KD) confirmed specificity of the anti-syntenin antibody (Fig. S1E). In agreement with the western blot analysis (Fig. 1G,H), *SMPD3* KD noticeably induced the intracellular accumulation of CD63, syntenin and lysobisphosphatidic acid (LBPA), an MVB-specific lipid marker (Fig. 2C,E,G). Accordingly, the total intracellular signal and the number of puncta per cell for these three markers were significantly increased following *SMPD3* KD (Fig. 2D,F,H). Collectively, these data suggest that reduced nSMase2 activity at the endosomal membrane results in intracellular accumulation of MVBs.

### nSMase2 regulates sEV secretion by modulating endolysosomal acidification

**Fig. 3.**
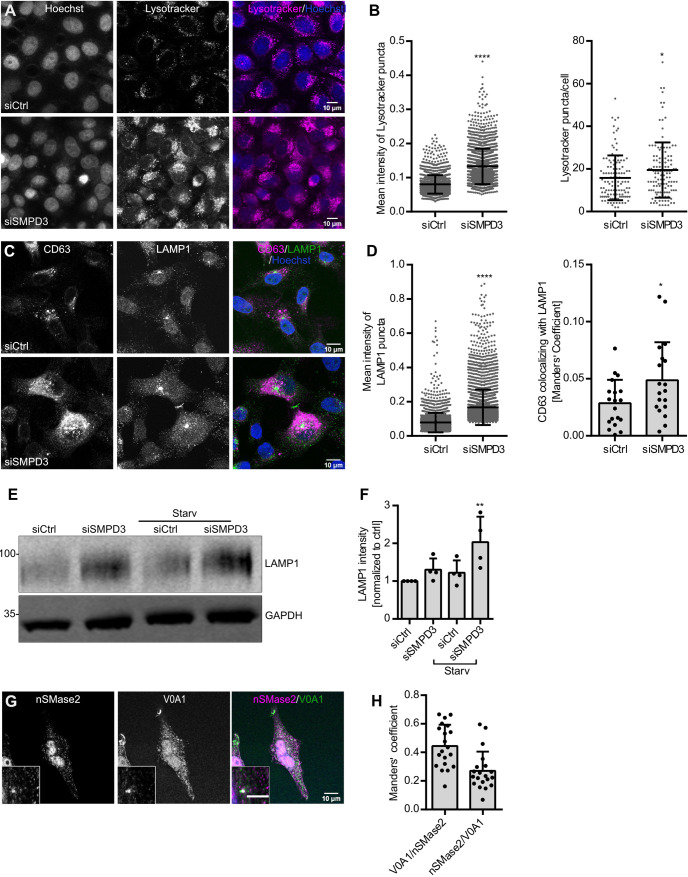
**nSMase2 regulates sEV secretion by modulating endosomal acidification.** (A) Confocal microscopy images of control (siCtrl) and *SMPD3* KD (siSMPD3) HeLa cells with intracellular acidic compartments labeled by Lysotracker. Nuclei were stained with Hoechst. (B) Quantifications of mean intensity of Lysotracker puncta (left) and Lysotracker puncta per cell (right) from A. Data are presented as mean±s.d.; *n*>50 cells from three biological replicates. *P<0.01; ****P<0.0001 (unpaired two-tailed Student's *t*-test). (C) Confocal microscopy images of control and *SMPD3* KD HeLa cells co-stained for the exosomal and MVB marker CD63 and the lysosomal marker LAMP1. Nuclei were stained with Hoechst. (D) Quantifications of mean fluorescence intensity of LAMP1 puncta (left) and colocalization analysis of CD63 with LAMP1 (right) from C. Data are presented as mean±s.d.; *n*=20 cells from two biological replicates. *P<0.01; ****P<0.0001 (unpaired two-tailed Student's *t*-test). (E) Western blot analysis of LAMP1 and GAPDH from control and *SMPD3* KD HeLa cells under normal and starvation (serum-depleted) conditions (Starv). Molecular masses are indicated in kDa. (F) Quantifications of LAMP1 signal normalized to loading control GAPDH before normalization to the control condition from E. The data shown in F represent the mean±s.d. of four biological replicates. **P<0.001 (one-way ANOVA followed by Dunnett's comparison test). (G) Representative confocal microscopy of HeLa cells co-stained for nSMase2 and ATP6V0A1 (V0A1). The scale bar in the magnified inset represents 5 µm. (H) Pixel-based colocalization between nSMase2 and V0A1 from G. Strong nSMase2 nuclear signal was excluded from the quantification by masking based on Hoechst staining. Data are presented as mean±s.d. of *n*>20 cells from two biological replicates.

Recently, several studies have shown that endosomal acidification plays a role in regulating MVB sorting. MVBs with low pH are targeted for lysosomal degradation, whereas MVBs with relatively higher pH are transported towards the cell periphery for plasma membrane fusion to release sEVs (Guo et al., 2017; Latifkar et al., 2019). As expected, bafilomycin A1 (Baf), which increases endolysosomal acidification by inhibiting V-ATPase activity, significantly increased the secretion of CD63, syntenin and CD81 in the sEV fraction (Fig. S1G,H) without significantly affecting the cell viability (Fig. S1F). NTA analysis further validated the increased sEV secretion upon Baf treatment (Fig. S1I,J). Based on these data, we next analyzed endolysosomal acidification upon *SMPD3* knockdown by staining with Lysotracker, a fluorescent dye that stains acidic cellular compartments (Robinson et al., 2012). We found that intracellular Lysotracker staining, as well as the number of acidic vesicles per cell, was significantly increased by *SMPD3* KD (Fig. 3A,B) or GW4869 treatment (Fig. S2A,B). Lysotracker staining was completely abolished by Baf treatment (Fig. S2C), thus confirming the staining specificity.

To further investigate how increased endolysosomal acidification upon *SMPD3* KD affects lysosomes and the trafficking of MVBs towards the lysosome, we next analyzed the intracellular colocalization of LAMP1 and CD63. *SMPD3* KD led to an increased colocalization between CD63 and LAMP1 compared with that in control cells (Fig. 3C,D). Interestingly, *SMPD3* KD significantly increased the LAMP1 signal intensity (Fig. 3C,D), which was further confirmed by western blot (Fig. 3E,F). The LAMP1 increase upon *SMPD3* KD was even more evident under a nutrient-depleted condition where cellular lysosomal activity peaks for autophagy (Levine, 2007) (Fig. 3E,F). Overall, these results indicate that *SMPD3* regulates endolysosomal acidification and alters MVB trafficking.

**Fig. 4.**
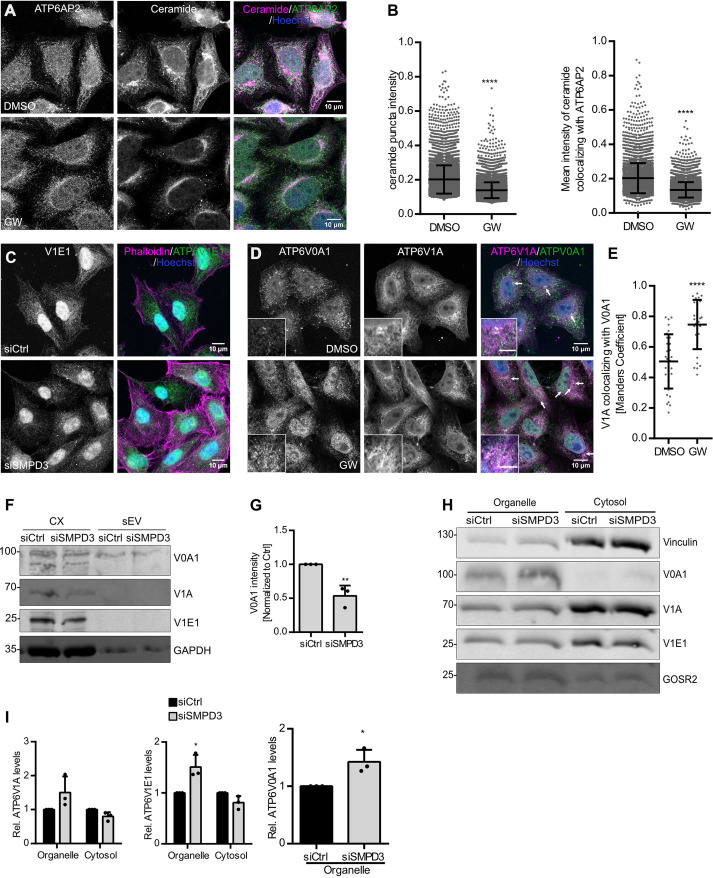
**nSMase2 regulates endolysosomal acidification by modulating V-ATPase assembly.** (A) Confocal microscopy images of HeLa cells treated with DMSO or GW4869 (GW), co-stained for ceramide and ATP6AP2. Nuclei were stained with Hoechst. (B) Dot plots of mean fluorescence intensity of ceramide puncta (left) and mean fluorescence intensity of ceramide that colocalized with ATP6AP2 (right) from A. Data are presented as mean±s.d.; *n*>50 cells from two biological replicates. ****P<0.0001 (unpaired two-tailed Student's *t*-test). (C) Confocal microscopy images of the cytoplasmic distribution of ATP6V1E1 (V1E1) in HeLa cells upon *SMPD3* KD (siSMPD3) compared with that of control cells (siCtrl). Actin was stained using phalloidin, and nuclei were stained using Hoechst. Images are representative of three experiments. (D) Confocal microscopy images of DMSO- and GW4869-treated HeLa cells co-stained for ATP6V0A1 and ATP6V1A. Nuclei were stained with Hoechst. Arrows indicate ATP6V0A1 and ATPV1A colocalization. The scale bar in the magnified inset represents 5 μm. (E) Dot plots showing colocalization analysis of ATP6V1A with ATP6V0A1 from D. Data are presented as mean±s.d.; *n*>20 from two biological replicates. ****P<0.0001 (unpaired two-tailed Student's *t*-test). (F) Western blot analysis of ATP6V0A1 (V0A1), ATP6V1A (V1A) and ATP6V1E1 (V1E1), as well as GAPDH as loading control, in total cell lysates (CX) and sEV fractions prepared from control (siCtrl) and *SMPD3* KD (siSMPD3) HeLa cells. (G) Quantification of signal intensity of V0A1 in sEV fractions from F normalized to the corresponding loading control GAPDH in the CX before normalization to the respective control. Data are presented as mean±s.d. of three biological replicates. **P<0.001 (unpaired two-tailed Student's *t*-test). (H) Western blot analysis of V0A1, V1A and V1E1 levels in organelle or cytosolic fractions upon *SMPD3* KD in HeLa cells. Vinculin and GOSR2 were used as cytosolic or organelle markers, respectively. (I) Quantifications of signal intensity of V1A and V1E1 in organelle and cytosolic fractions, and V0A1 in the organelle fraction from H. Cytosolic and organelle signals for each subunit were normalized to their respective loading control, vinculin or GOSR2, before normalization to the control condition. Data are presented as mean±s.d. of three biological replicates. *P<0.01 (left, unpaired two-tailed Student's *t*-test; middle and right, one-way ANOVA followed by Sidak's multiple comparisons test). Molecular masses in D and H are indicated in kDa.

The V-ATPase complex acidifies endosomes and lysosomes by translocating protons into their lumina in an ATP-dependent manner (Wang et al., 2021). As we found nSMase2 activity to be reduced at HRS-positive endosomes (Fig. 2A,B) and acidification increased upon *SMPD3* KD (Fig. 3A,B), we next tested whether nSMase2 and V-ATPase reside on the same endosomes. Indeed, a small fraction of nSmase2 colocalized with ATP6V0A1, a V0 transmembrane subunit of V-ATPase, in punctate structures in immunostaining (Fig. 3G,H). This is in line with an affinity-purification-based mass spectrometry study that showed nSmase2 interaction with ATP6V0A1 (Huttlin et al., 2017). Moreover, the mean intensity of ceramide puncta colocalizing with transmembrane ATP6AP2, a core V-ATPase V0 subunit, was significantly reduced by GW4869 treatment, confirming nSMase2 activity on V-ATPase-positive membranes (Fig. 4A,B). Therefore, these data indicate that nSMase2 and V-ATPase activity could be interlinked on MVBs to regulate their acidification for sEV secretion.

### nSMase2 regulates endolysosomal acidification by modulating V-ATPase assembly

V-ATPase complexes on endosomal membranes are partitioned into raft-like domains, enriched in sphingomyelin and cholesterol (Lafourcade et al., 2008), and their activity is regulated by the reversible assembly of the complex. For example, detachment of the cytoplasmic V1 domain, ATP6V1E1 subunit, from the V-ATPase complex has been shown to decrease its activity in yeast and mammalian cells (Sautin et al., 2005; Toei et al., 2010). A recent study has unraveled that ATG5 detaches ATP6V1E1 from the complex to deacidify the MVB lumen, thereby resulting in increased exosome secretion (Guo et al., 2017). We found that ATP6V1E1 localization changed from a diffuse pattern in the cytoplasm in control cells to a more punctate vesicular structure in *SMPD3* KD cells (Fig. 4C). Parallel to the findings of the study mentioned above, this could indicate an increase in ATP6V1E1 localization to endosomes, for example to MVBs. To analyze whether inhibition of nSMase2 activity increases endolysosomal acidification by inducing V-ATPase complex assembly, we analyzed the colocalization between ATP6V0A1 and ATP6V1A. Indeed, nSMase2 activity inhibition using GW4869 significantly increased the colocalization between ATP6V0A1 and ATP6V1A, indicating increased V-ATPase assembly (Fig. 4D,E). Therefore, we next analyzed whether V-ATPase was internalized into ILVs during MVB maturation and subsequently secreted on sEVs. Indeed, under control conditions, we found the transmembrane subunit ATP6V0A1 secreted in the sEV fraction, possibly confirming that it can be internalized into ILVs and released into sEVs. Interestingly, nSMase2 activity inhibition using GW4869 reduced this secretion. ATP6V1A and ATP6V1E1, two cytosolic subunits of the V-ATPase complex, were absent from the sEV fraction under both conditions (Fig. 4F,G). The total intracellular levels of ATP6V0A1, ATP6V1E1, and ATP6V1A in total RIPA lysates were unaffected by *SMPD3* KD (Fig. S2D,E). These data show that ATP6V0A1 is secreted on sEVs in a nSMase2-dependent manner. To further confirm that *SMPD3* KD stabilizes the V-ATPase complex on the endosomal membrane, we next performed a biochemical cell fractionation. This assay yielded a cytosolic and an organelle fraction, the latter containing the membrane proteins (Itzhak et al., 2016). *SMPD3* KD indeed enriched ATP6V1E1 and ATP6V0A1 in the organelle fraction in comparison to control cells (Fig. 4H,I), suggesting increased assembly of V-ATPase complex at the membrane. Taken together, our data show that nSMase2 regulates sEV secretion by modulating V-ATPase assembly and activity on MVBs.

### MVB cholesterol levels regulate sEV secretion by modulating V-ATPase assembly

**Fig. 5.**
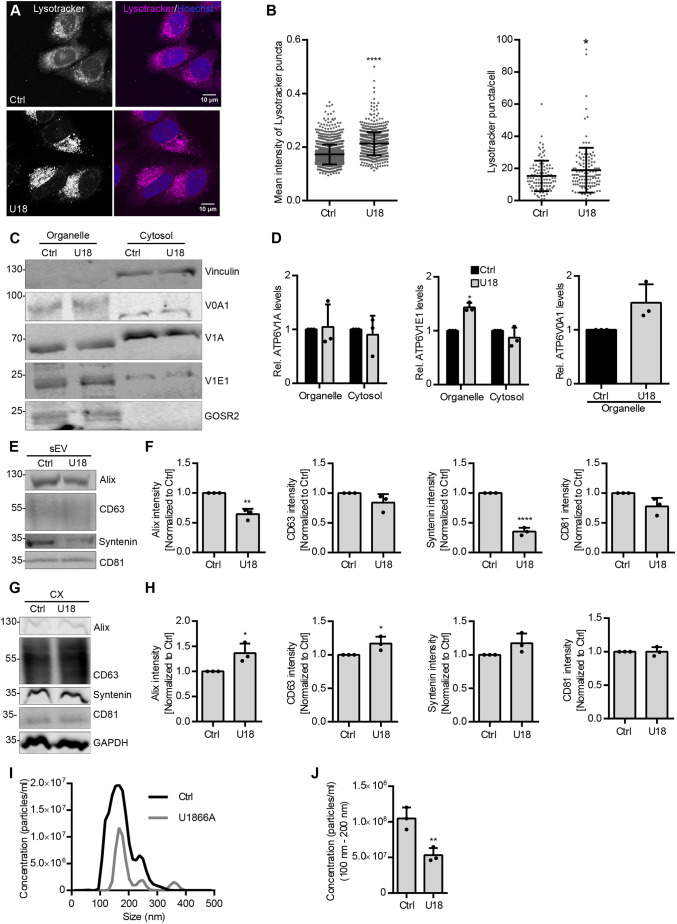
**MVB cholesterol levels regulate sEV secretion by modulating V-ATPase assembly.** (A) Confocal microscopy images of control (Ctrl) and U18666A (U18)-treated HeLa cells with intracellular acidic compartments labeled by Lysotracker. Nuclei were stained with Hoechst. (B) Quantifications of mean intensity of Lysotracker puncta (left) and Lysotracker staining puncta per cell from A. Data are presented as mean±s.d.; *n*>50 cells from three biological replicates. *P<0.01; ****P<0.0001 (unpaired two-tailed Student's *t*-test). (C) Western blot analysis of ATP6V0A1 (V0A1), ATP6V1A (V1A) and ATP6V1E1 (V1E1) levels in organelle or cytosolic fractions upon U18 treatment in HeLa cells. Vinculin and GOSR2 were used as the cytosolic and organelle markers, respectively. (D) Quantifications of signal intensity of V1A and V1E1 in organelle and cytosolic fractions, and V0A1 in the organelle fraction from C. Cytosolic and organelle signals for each subunit were normalized to their respective loading control, vinculin or GOSR2, before normalization to the control condition. (E) Western blot analysis of Alix, CD63, syntenin and CD81 in sEV fractions prepared from equal amounts of control or U18-treated HeLa cells. (F) Signal intensity quantifications of Alix, CD63, syntenin and CD81 in sEV fractions from E, calculated by normalizing the signal to loading control GAPDH levels in the corresponding cell lysates in G before normalization to their respective control. (G) Western blot analysis of the indicated exosomal markers in corresponding cell lysates (CX) from E. GAPDH was probed as a loading control. (H) Quantifications of intracellular Alix, CD63, syntenin and CD81 signal intensity from G normalized to loading control GAPDH before normalization to the respective control. (I) Representative size distribution of sEVs isolated from control and U18-treated HeLa cells. (J) NTA quantification of sEV concentration from I. The data shown in D,F,H and J represent the mean±s.d. of three biological replicates. *P<0.01; **P<0.001 (unpaired two-tailed Student's *t*-test except for middle and right graphs in D where a one-way ANOVA with Sidak's multiple comparisons test was used). Molecular masses in C,E and G are indicated in kDa.

As nSMase2 hydrolyzes sphingomyelin into ceramide and phosphorylcholine, and ceramide triggers ILV formation, we reasoned that the enrichment of sphingomyelin on endosomal membranes upon *SMPD3* KD could support the assembly of V-ATPase complexes for acidification. Sphingomyelin forms lipid-ordered microdomains with cholesterol in model and cellular membrane systems (Hullin-Matsuda et al., 2014). Different transmembrane protein complexes, such as V-ATPases, are preferentially sorted into these microdomains, allowing their structural and functional regulation (Sezgin et al., 2017). A recent study reported the complete cryo-EM structure of the human V-ATPase complex and revealed that ordered lipid molecules including cholesterol are an integral part of the V0 complex (Wang et al., 2020). Hence, we hypothesized that sphingomyelin and cholesterol levels at MVBs could modulate V-ATPase assembly and thereby regulate MVB fate and sEV secretion. Therefore, similar to the enrichment of sphingomyelin upon *SMPD3* KD, enrichment of cholesterol should increase endosomal acidification through supporting V-ATPase complex assembly, and at the same time reduce sEV secretion. To specifically modulate cholesterol at the MVB level, we used U18666A (U18), which accumulates cholesterol in MVBs by inhibiting the cholesterol transporter Nieman–Pick C1 (NPC1) (Lu et al., 2015). As expected, U18 treatment, with a mild (<5%) cell viability reduction (Fig. S2F), significantly increased mean Lysotracker staining as well as Lysotracker puncta per cell (Fig. 5A,B), indicating increased V-ATPase activity upon MVB cholesterol accumulation. Additionally, U18 enriched both the transmembrane subunit ATP6V0A1 and the cytoplasmic subunit ATP6V1E1 in the membrane fraction, similar to *SMPD3* KD (Fig. 5C,D). Accordingly, cholesterol accumulation also reduced the secretion of the exosomal markers Alix and syntenin in the sEV fraction (Fig. 5E,F) with a concomitant slight increase in the intracellular levels of these proteins (Fig. 5G,H). NTA analysis further validated the reduction of sEV secretion upon U18 treatment (Fig. 5I,J). These data demonstrate that, in addition to ceramide and sphingomyelin, endosomal cholesterol levels also modulate both V-ATPase activity and sEV secretion. This strengthens the idea that the endosomal lipid environment governs secretory versus degradative MVB sorting by modulating endosomal acidification via V-ATPase recruitment, thereby exerting control over sEV secretion levels.

### TNFα regulates sEV secretion through nSMase2 activation

**Fig. 6.**
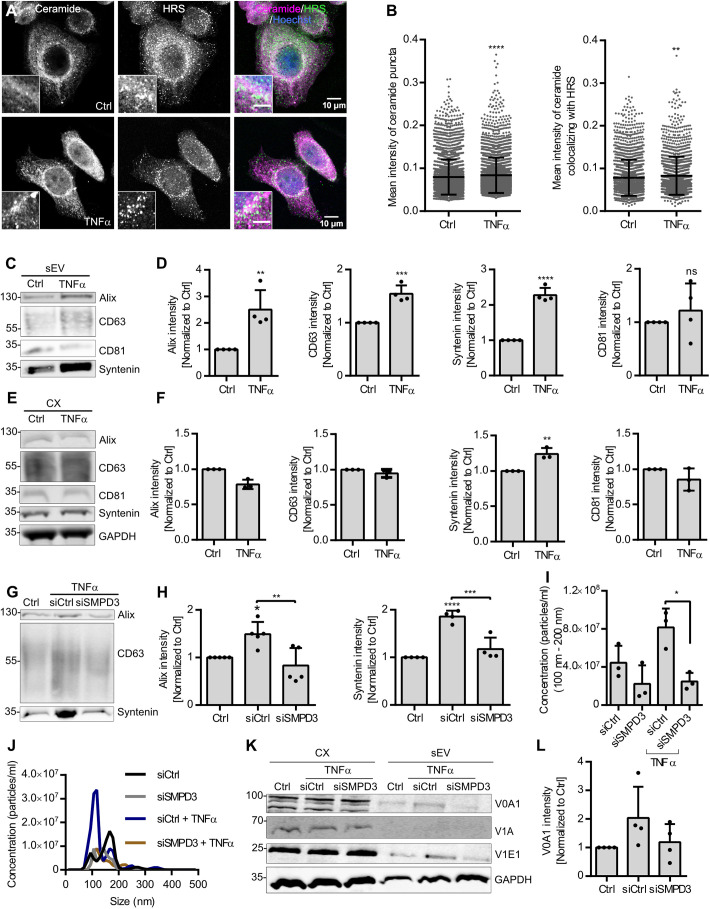
**TNFα regulates sEV secretion through nSMase2 activation.** (A) Confocal microscopy images of control (Ctrl) and TNFα-treated HeLa cells co-stained for ceramide and HRS. Nuclei were stained with Hoechst. The scale bar in the magnified inset represents 5 µm. (B) Dot plots of mean fluorescence intensity of ceramide puncta (left) and mean fluorescence intensity of ceramide that colocalized with HRS (right) from A. Data are presented as mean±s.d.; *n*>50 cells from two biological replicates. **P<0.001; ****P<0.0001 (unpaired two-tailed Student's *t*-test). (C) Western blot analysis of Alix, CD63, syntenin and CD81 in sEV fractions prepared from equal amounts of control or TNFα-treated HeLa cells. (D) Signal intensity quantifications of Alix, CD63, syntenin and CD81 in sEV fractions from C, calculated by normalizing the signal to loading control GAPDH levels in the corresponding cell lysates in E before normalization to their respective control. Data are presented as mean±s.d. of four biological replicates. **P<0.001; ***P<0.0004; ****P<0.0001; ns, not significant (unpaired two-tailed Student's *t*-test). (E) Western blot analysis of the indicated exosomal markers, GAPDH and calnexin in the corresponding cell lysates (CX) from C. GAPDH was probed as a loading control. (F) Quantifications of intracellular Alix, CD63, syntenin and CD81 signal intensity from E normalized to loading control GAPDH before normalization to the respective control. Data are presented as mean±s.d. of three biological replicates. **P<0.001 (unpaired two-tailed Student's *t*-test). (G) Western blot analysis of Alix, CD63 and syntenin in an sEV fraction prepared from an equal amount of control untreated cells (Ctrl) or control siRNA-treated (siCtrl) and *SMPD3* KD (siSMPD3) HeLa cells treated with TNFα. (H) Signal intensity quantifications of Alix and syntenin in sEV fractions from G, calculated by normalizing the signal to loading control GAPDH levels in the corresponding cell lysates before normalization to their respective control. Data are presented as mean±s.d. of five (Alix) and four (syntenin) biological replicates. *P<0.01, **P<0.001, ***P<0.0004, ****P<0.0001 (one-way ANOVA followed by Tukey's multiple comparisons test). (I) NTA quantification of sEV concentration isolated from control and *SMPD3* KD HeLa cells with or without TNFα treatment. Data are presented as mean±s.d. of three biological replicates. *P<0.01 (one-way ANOVA followed by Tukey's multiple comparisons test). (J) Representative size distribution of sEV analyzed by NTA from I. (K) Western blot analysis of ATPV0A1 (V0A1), ATP6V1A (V1A) and ATP6V1E1 (V1E1), as well as GAPDH as loading control, in the total cell lysates (CX) and corresponding sEV fractions prepared from untreated HeLa cells (Ctrl) and from control and *SMPD3* KD HeLa cells treated with TNFα. (L) Quantifications of signal intensity of V0A1 in the sEV fraction from K normalized to the corresponding loading control GAPDH in the CX before normalization to the respective control. Data are presented as mean±s.d. of four biological replicates. (one-way ANOVA followed by Tukey's multiple comparisons test). Molecular masses in C,E,G and K are indicated in kDa.

To investigate the functional implication of nSMase2 counteracting V-ATPase activity on endosomes, we investigated the possible role of TNFα upstream of nSMase2. TNFα is a critical pro-inflammatory cytokine implicated in the pathogenesis of various diseases such as cancer (reviewed in Balkwill, 2006). In addition to its role in the regulation of immunogenic reactions, TNFα mediates different cellular processes, including cellular proliferation, differentiation and migration (Balkwill, 2006). Among its pleiotropic actions, TNFα activates nSmase2 by translocating polycomb group protein EED from the nucleus. This translocation of the EED allows the recruitment of nSMase2 to the TNF receptor-1 (TNF-R1)–FAN–RACK1 complex for its activation (Philipp et al., 2010). In line with these data, we found increased ceramide staining upon TNFα treatment compared with that in the control cells, as well as increased ceramide puncta intensity colocalizing with HRS (Fig. 6A,B), these data indicate that TNFα activates nSMase2 on the endosomal membranes. Even though the number of acidic puncta per cell was increased, TNFα treatment, with less than 10% reduction of cell viability (Fig. S2G), significantly decreased the total Lysotracker signal intensity, thereby indicating reduced V-ATPase activity (Fig. S2H,I).

Following the idea that TNFα might redirect MVBs towards secretion through its effect on nSMase2 and V-ATPase activity, we next investigated the effects of TNFα on sEV secretion directly. TNFα treatment indeed significantly increased the secretion of the exosomal markers Alix, CD63 and syntenin in the sEV fraction (Fig. 6C,D). While intracellular syntenin levels were significantly upregulated by TNFα treatment, no effect was observed for the intracellular levels of the exosomal markers CD63, Alix and CD81 (Fig. 6E,F). Importantly, the increased secretion of Alix, CD63 and syntenin in the sEV fraction induced by TNFα in control cells were rescued by *SMPD3* KD (Fig. 6G,H; Fig. S2J). NTA analysis of sEV samples further confirmed that increased sEV secretion by TNFα was rescued by *SMPD3* KD (Fig. 6I,J). confirming that TNFα stimulation affects sEV secretion via nSMase2 activity.

**Fig. 7.**
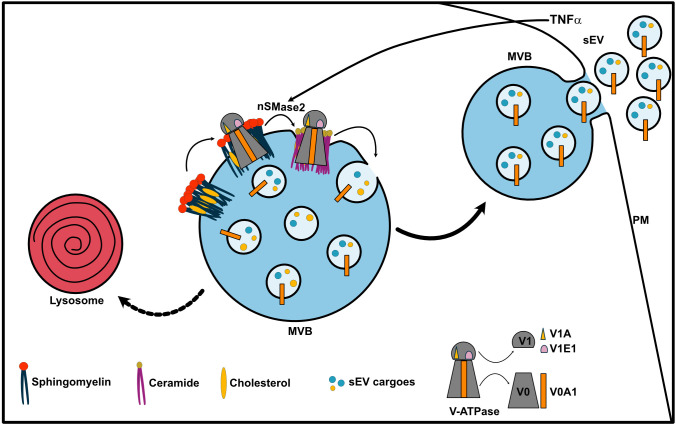
**nSMase2 regulates sEV secretion by counteracting V-ATPase-mediated endosomal acidification.** Working model showing how nSMase2 regulates sEV secretion via counteracting V-ATPase-mediated endosome acidification. Ceramide generated by nSMase2 activity on the MVB drives inward membrane invagination to selectively incorporate the V-ATPase subunit ATP6V0A1 (V0A1) and other sEV cargoes into ILVs targeted for secretion. The sequestration of V0A1 into ILVs attenuates MVB lumen acidification to favor secretory MVB trafficking for sEV release. V1A, ATP6V1A; V1E1, ATP6V1E1.

In alignment with our hypothesis that TNFα acts through nSMase2 activation at the MVB membrane and subsequently promotes V-ATPase sequestration into the ILV, TNFα furthermore increased ATP6V0A1 levels in the sEV fraction (Fig. 6K,L). Concordantly, *SMPD3* KD partially rescued the increased ATP6V0A1 secretion on sEVs (Fig. 6K,L). Collectively, these results indicate that TNFα activates nSMase2 on endosomes, which in turn counteracts V-ATPase activity on the MVB membrane and reduces endosomal acidification to promote sEV secretion (Fig. 7).

## DISCUSSION

Here, we demonstrate that nSMase2 controls exosome secretion by counteracting V-ATPase activity on endosomal membranes. We show a previously unknown mechanism by which nSMase2 regulates sEV secretion and provide evidence that the lipid environment at MVBs, and specifically the levels of ceramide, sphingomyelin and cholesterol, regulate sEV secretion by modulating endosomal acidification. Importantly, we show that TNFα, a prominent pro-inflammatory cytokine known to activate nSMase2, promotes sEV secretion by modulating endosomal acidification via changes in V-ATPase complex assembly. These findings for the first time establish a molecular connection between TNFα-induced nSMase2 activation and sEV secretion.

Owing to its biophysical properties, ceramide generated by nSMase2 at the MVB membrane drives inward membrane budding to form ILVs (Trajkovic, 2008). We show that this impaired MVB membrane invagination affects sEV secretion by increasing V-ATPase activity through stabilizing it on the endosomal membrane. We propose that V-ATPases are selectively sequestered into MVBs under normal conditions to attenuate their lumen acidification activity and promote secretory MVB trafficking. We found that nSMase2 depletion reduces the sequestration of V-ATPase subunits into ILVs and instead renders them active on the MVB membrane for continued acidification. This consequently deregulates secretory MVB sorting and therefore reduces sEV secretion. Sequestration of growth factor receptors bound to their ligands into ILVs has been shown to attenuate their signaling by targeting them for lysosomal degradation (Piper and Katzmann, 2010). Conversely, ILV sequestration of glycogen synthase kinase β (GSKβ, encoded by *GSK3B*), triggered by WNT signaling, stabilizes many GSKβ protein substrates which are otherwise targeted for degradation (Taelman et al., 2010). We show that V-ATPase sequestration into ILVs occurs in a ceramide-dependent manner that significantly affects MVB trafficking fate and sEV secretion.

Recent studies have highlighted the role of MVB lumen acidification as a sorting signal – MVBs with less acidic lumen are sorted as secretory MVBs for exosome release. For example, ATG5 and LC3, independent of canonical macroautophagy, coordinately deacidify MVBs to promote sEV secretion (Guo et al., 2017). Additionally, numerous studies have reported that exosome secretion is controlled by endolysosomal acidification (Villarroya-Beltri et al., 2016; Edgar et al., 2016). Our findings on how changes in nSMase2-mediated endosomal acidification affect sEV secretion further substantiate these findings.

In addition to ceramide and sphingomyelin, we find MVB cholesterol levels to regulate sEV secretion through modulation of V-ATPase-mediated endosomal acidification. Cholesterol, in association with sphingomyelin, forms lipid-ordered microdomains on the membrane, providing an assembly platform for different protein complexes. This establishes a functional and structural connection between lipids and proteins, possibly allowing for a reciprocal regulation. The complete cryo-EM structure of the human V-ATPase complex has revealed that ordered lipid molecules including cholesterol are indeed an integral part of the V0 complex (Wang et al., 2020). Cholesterol, together with ceramide generated by nSMase2 activity on the MVB membrane, may invaginate with V-ATPase subunits to form ILVs, which are known to harbor the highest cholesterol content in the endocytic pathway (Möbius et al., 2003).

The reported cell type-specific effects of nSMase2 inhibition on sEV secretion may be explained by the differences in cellular endosomal acidification levels, which in turn could be affected by different cellular and physiological factors. ESCRT components drive ILV biogenesis at the MVB (Saksena et al., 2007), and knockdown of the ESCRT components HRS, STAM1 and TSG101 reduces the secretion of CD63-positive EVs (Colombo et al., 2013). However, the role of the ESCRT complexes in cargo loading and ILV generation for exosome secretion is increasingly challenged by other studies. For example, a recent study has shown that knockdown of individual ESCRT subunits counterintuitively increases exosome secretion by causing lysosomal dysfunction (Matsui et al., 2021). These findings are also in line with studies showing that the lysosomal degradation of several endocytosed receptors requires the ESCRT machinery for proper internalization of these receptors into ILVs (Futter et al., 1996; Haglund et al., 2003; Yamashita et al., 2008). Therefore, based on these studies and our results, it is tempting to speculate that ESCRT complexes support ILV generation for degradative MVBs that are targeted for lysosomes. On the other hand, ceramide generated by nSMase2 drives membrane invagination on the MVB to package V-ATPases into ILVs, thereby attenuating their lumen acidification activity and favoring secretory MVB transport for sEV release. These two pathways involving ceramide or the ESCRT machinery provide two mechanisms of ILV formation that have different outcomes for the fate of their endosomal compartment.

TNFα, a pro-inflammatory cytokine, activates nSMase2. TNFα activates TNF-R1, which translocates EED from the nucleus. EED then simultaneously interacts with RACK1 and nSMase2. This interaction couples EED and nSMase2 to the TFN-R1–FAN–RACK1 complex and activates nSMase2 (Philipp et al., 2010). However, the exact downstream effect of the TNFα-induced nSMase2 pathway on cellular processes is not fully understood. Our findings elucidate the mechanism by which TNFα-induced nSMase2 activation promotes sEV secretion by modulating endosomal acidification. TNFα stimulation has been shown to compromise lysosomal integrity, which affects the cellular degradative capacity and reduces autophagic flux (Wang et al., 2015; Werneburg et al., 2002). The exact molecular mechanism underlying this process remains unknown. Based on our findings, it would be worthwhile to investigate whether this occurs via nSMase2 activation.

In addition to the proposed role of nSMase2 in the initial ILV generation (Trajkovic et al., 2008), we show that nSMase2 activity controls later MVB trafficking by counteracting V-ATPase-mediated lumen acidification, thereby promoting a secretory rather than a degradative fate. With increased lysosome biogenesis upon *SMPD3* KD under starvation conditions (Fig. 3C–F), how nSMase2-mediated endosomal acidification regulation affects autophagy remains an interesting question to explore. Furthermore, how nSMase2-dependent MVB deacidification affects recruitment of further MVB secretory machinery including Rab27a, Rab27b (Ostrowski et al., 2010) and SNARE proteins such as YKT6 (Gross et al., 2012) remains to be investigated. Further study on TNFα-induced nSMase2 activation in cancer cells may shed light on the role of TNFα and sEV in cancer progression.

## MATERIALS AND METHODS

### Cell culture and transfection

HeLa cells (kindly provided by Holger Bastians, University of Goettingen, Germany) were maintained in DMEM (Thermo Fisher Scientific Life Technologies, 52100021) supplemented with 10% fetal calf serum (Biochrom) and 10 μg/ml penicillin-streptomycin (Sigma Aldrich, P4333) at 37°C in a humidified atmosphere with 5% CO_2_. Cells were authenticated and checked for mycoplasma contamination on a regular basis. Cells were transiently transfected with Screenfect siRNA (Dharmacon) for 72 h according to the manufacturer's instructions. Cells were treated with the following drugs for 16 h:GW4869 (5 μM; Sigma Aldrich, D1692), bafilomycin A1 (100 ng/ml; Sigma Aldrich, B1793), TNFα (5 ng/ml; Peprotech, 300-01A) and U18666A (10 µM; Sigma Aldrich, U3633). The Dharmacon SMARTpool siRNAs used to target *SMPD3* were as follows: D-006678-01, siSMPD3 #1, 5′-CAACAGCGGCCUCCUCUUU-3′; D-006678-04, siSMPD3 #2, 5′-CAAGCGAGCAGCCACCAAA-3′; D-006678-17, siSMPD3 #3, 5′-ACCAAAGAAUCGUCGGGUA-3′; D-006678-18, siSMPD3 #4, 5′-CGAACGGCCUGUACGAUGA-3′. The control non-targeting siRNA (siCtrl; D-001210-03, Dharmacon) was 5′-AUGUAUUGGCCUGUAUUAG-3′. The siRNA used to target ubiquitin (siUBC; D-19408-01, Dharmacon) was 5′-GUGAAGACCCUGACUGGUA-3′.

### Antibodies

The following antibodies and dilutions were used for western blotting (WB) or for immunofluorescence staining (IF): Alix (WB 1:1000; Santa Cruz, sc53540), CD63 (WB 1:100; IF 1:10; H5C6, AB_528158, Developmental Studies Hybridoma Bank), syntenin (WB 1:1000; IF 1:100; Abcam, ab133267), GAPDH (WB 1:1000; Millipore, CB1001), Calnexin (WB 1:1000; IF 1:100; Abcam, ab75801), HRS (IF 1:100; ProteinTech, 1039-1-AP), ceramide (IF 1:100; Enzo, ALX-804-196), LBPA (IF 1:100; Millipore, MABT837), nSMase2 (IF 1:100; Santa Cruz, sc-166637), ATP6V0A1 (WB 1:1000; IF 1:100; Novus Bio, NB1-89342), vinculin (WB 1:1000; ProteinTech, 66305-1-Ig), ATP6V1E1 (WB 1:1000; IF 1:100; ProteinTech, 15280-1-AP), ATP6V1A (WB 1:1000; Abnova, H00000523-M02), GOSR2 (WB 1:1000 ProteinTech, 66134-1-Ig), LAMP1 (WB 1:1000; IF 1:100; Abcam, ab24170).

### Small extracellular vesicle isolation

For sEV isolation, 400,000 cells (siRNA transfection) or 500,000 cells (inhibitor treatment) were seeded. sEVs were purified by differential centrifugation as previously described (Menck et al., 2017). In short, cells were incubated with an sEV-free growth medium for defined periods. Subsequently, the conditioned medium was collected and subjected to sequential centrifugation steps at 750 ***g***, 1500 ***g*** and 14,000 ***g***, before pelleting sEVs at 100,000 ***g***. The sEV pellet was lysed with RIPA lysis buffer [50 mM Tris-HCl (pH 7.5), 150 mM NaCl, 1% (v/v) Igepal, 0.5% (w/v) SDS, 1x Roche protease inhibitor] diluted at 1:1 with phosphate-buffered saline (PBS).

### Western blot analysis

Cell and sEV lysates, in SDS–PAGE sample buffer (300 mM Tris-HCl pH 6.8, 12% SDS, β-mercaptoethanol), were boiled for 5 min before the proteins were separated on 4–12% gradient gels (Bolt Bis-Tris Plus Gels, Thermo Fisher Scientific). Proteins were then transferred to PVDF membranes (Merck) and blocked with 5% (w/v) milk in Tris-buffered saline containing Tween 20 (TBST; 20 mM Tris-HCl pH 8.0, 75 mM NaCl, 0.2% Tween-20) for 30 min. Membranes were incubated with primary antibodies overnight at 4°C. After washing, membranes were incubated with fluorescently labeled secondary antibodies at room temperature in the dark and detected using the Li-COR Odyssey system. Quantitative measurements were done with Fiji ImageJ (NIH, Bethesda, MD).

### Nanoparticle tracking analysis

A Malvern Panalytical Nanosight NS300 instrument was used to measure sEV particles diluted in PBS (1:100) using the parameters camera level 14, screen gain 10.8, detection threshold 5. For each sample, a total of three videos of 30–60 s was measured. The videos were analyzed using the NanoSight NTA 2.3 Analytical Software, and the particle concentration, size distribution, and the general mean and mode of the samples were obtained.

### Immunostaining, microscopy and image analysis

Cells grown in 8-well microscopic coverslips (Sarstedt, 94.6170.802), reverse transfected with siRNAs or treated as indicated, were fixed and permeabilized with 4% paraformaldehyde and 0.2% Triton X-100. The slides were then blocked in 3% bovine serum albumin diluted in PBS, followed by 90 min incubation with primary antibodies. After washing three times, cells were incubated with secondary antibodies conjugated to Alexa Fluor-488 or -546. Nuclei and actin were labeled using Hoechst 33342 (Hoechst; Thermo Fisher Scientific, 62249) and conjugated phalloidin, respectively. Live cells were incubated with 200 nM Lysotracker DND-red-99 (Thermo Fisher Scientific Life Technologies, L7528) for 45 min to label acidic compartments and fixed with 4% paraformaldehyde after washing three times with PBS. The cells were visualized with a Zeiss LSM780 confocal microscope (Plan Neofluor 63×/oil NA 1.4 objective). Staining and microscopy conditions were kept identical for comparisons. Mean fluorescence intensity of puncta, the number of puncta per cell and colocalization quantifications were performed using available pipelines with some modifications in CellProfiler (Broad Institute of MIT and Harvard).

### Viability assay

Cell viability was measured by performing a CellTiter-Glo assay (Promega, G8461). Cells were seeded in a 96-well plate. After the indicated treatment, 100 µl of the CellTiter-Glo reagent (Promega) was added to each well (1:1). The plate was incubated on a shaker for 2 min at room temperature to allow cell lysis and then incubated without shaking for 10 min to allow luminescence signal stabilization. The signal was measured using a luminometer, and data were analyzed using MikroWin 2000 lite Version 4.43.

### Real-time qPCR

Total RNA was isolated from cells using Trizol (Thermo Fisher Scientific Invitrogen, 15596026). Equal amounts of RNA (2 μg) were reverse transcribed into cDNA by treatment with M-MLV reverse transcriptase (Invitrogen, 28025013). The resulting cDNA product was analyzed by real-time quantitative PCR using iTaq Universal SYBRgreen Supermix (Bio-Rad, 172-5125) and gene-specific primers (β-actin forward, 5′-GAGCACAGAGCCTCGCCTTT-3′; β-actin reverse, 5′-ACATGCCGGAGCCGTTGTC-3′; *SMPD3* forward, 5′-CAACAAGTGTAACGACGATGCC-3′; *SMPD3* reverse, 5′-CGATTCTTTGGTCCTGAGGTGT-3′). Transcript Ct values were converted to fold change expression changes (2^−ΔΔCt^ values) after normalization to the housekeeping gene β-actin. Quantitative real-time PCR was performed using the CFX system (Bio-Rad).

### Organelle and membrane fractionation

Cells were fractionated into organelle and cytosolic fractions based on a published protocol (Itzhak et al., 2016). Briefly, all steps were performed on ice with pre-chilled ice-cold buffer. Cells were incubated in hypotonic lysis buffer (25 mM Tris-HCl, pH 7.5, 50 mM sucrose, 0.5 mM MgCl_2_, 0.2 mM EGTA) for 5 min after washing once with PBS and once with hypotonic lysis buffer. Cells were then scraped in fresh hypotonic lysis buffer and transferred to a Dounce homogenizer (B. Braun) and homogenized with 15 strokes with the tight pestles (B. Braun). Sucrose concentration was immediately restored to 250 mM with hypertonic sucrose buffer (2.5 M sucrose, 25 mM Tris-HCl pH 7.5, 0.5 mM MgCl_2_, 0.2 mM EGTA) after homogenization. Homogenized crude cell lysates were centrifuged at 1000 ***g*** for 10 min to separate nuclear material. The organelle fraction was obtained by centrifuging the post-nuclear supernatant at 78,400 ***g*** for 30 min, and the resulting supernatant was collected as the cytosolic fraction. The organelle pellet was dissolved in SDS buffer (2.5% SDS, 50 mM Tris-HCl pH 8.1) and heated for 5 min at 72°C.

### Protein concentration determination

Protein concentration was determined by using the Pierce BCA Protein Assay Kit (Thermo Fisher Scientific, 23225) according to the manufacturer's protocol. Protein lysates diluted with PBS were added to 500 µl of a 1:50 mixture of Buffer B and Buffer A. The mixture was incubated at 60°C for 30 min and transferred to cuvettes for analysis with a NanoDrop. A standard curve for protein BCA analysis and PBS as blank were used.

### Statistics

Data were analyzed using GraphPad Prism 6 built-in tests. All data are presented as means±s.d. Details of the significance tests, the number of replicates and the *P* values are reported in the figure legends.

## Supplementary Material

Supplementary information

Reviewer comments

## References

[JCS259324C1] Baietti, M. F., Zhang, Z., Mortier, E., Melchior, A., Degeest, G., Geeraerts, A., Ivarsson, Y., Depoortere, F., Coomans, C., Vermeiren, E. et al. (2012). Syndecan-syntenin-ALIX regulates the biogenesis of exosomes. *Nat. Cell Biol.* 14, 677-685. 10.1038/ncb2502 22660413

[JCS259324C2] Balkwill, F. (2006). TNF-α in promotion and progression of cancer. *Cancer Metastasis Rev.* 25, 409-416. 10.1007/s10555-006-9005-3 16951987

[JCS259324C3] Bebelman, M. P., Smit, M. J., Pegtel, D. M. and Baglio, S. R. (2018). Biogenesis and function of extracellular vesicles in cancer. *Pharmacol. Ther.* 188, 1-11. 10.1016/j.pharmthera.2018.02.013 29476772

[JCS259324C4] Ciardiello, C., Cavallini, L., Spinelli, C., Yang, J., Reis-Sobreiro, M., De Candia, P., Minciacchi, V. R. and Di Vizio, D. (2016). Focus on extracellular vesicles: New frontiers of cell-to-cell communication in cancer. *Int. J. Mol. Sci.* 17, 1-17. 10.3390/ijms17020175 PMC4783909 26861306

[JCS259324C5] Colombo, M., Moita, C., Van Niel, G., Kowal, J., Vigneron, J., Benaroch, P., Manel, N., Moita, L. F., Théry, C. and Raposo, G. (2013). Analysis of ESCRT functions in exosome biogenesis, composition and secretion highlights the heterogeneity of extracellular vesicles. *J. Cell Sci.* 126, 5553-5565. 10.1242/jcs.128868 24105262

[JCS259324C6] Danzer, K. M., Kranich, L. R., Ruf, W. P., Cagsal-Getkin, O., Winslow, A. R., Zhu, L., Vanderburg, C. R. and McLean, P. J. (2012). Exosomal cell-to-cell transmission of alpha synuclein oligomers. *Mol. Neurodegener* 7, 1-18. 10.1186/1750-1326-7-42 22920859 PMC3483256

[JCS259324C7] Edgar, J. R., Manna, P. T., Nishimura, S., Banting, G. and Robinson, M. S. (2016). Tetherin is an exosomal tether. *Elife* 5, 1-19. 10.7554/eLife.17180 PMC5033606 27657169

[JCS259324C8] Forgac, M. (2007). Vacuolar ATPases: rotary proton pumps in physiology and pathophysiology. *Nat. Rev. Mol. Cell Biol.* 8, 917-929. 10.1038/nrm2272 17912264

[JCS259324C9] Futter, C. E., Pearse, A., Hewlett, L. J. and Hopkins, C. R. (1996). Multivesicular endosomes containing internalized EGF-EGF receptor complexes mature and then fuse directly with lysosomes. *J. Cell Biol.* 132, 1011-1023. 10.1083/jcb.132.6.1011 8601581 PMC2120766

[JCS259324C10] Gross, J. C., Chaudhary, V., Bartscherer, K. and Boutros, M. (2012). Active Wnt proteins are secreted on exosomes. *Nat. Cell Biol.* 14, 1036-1045. 10.1038/ncb2574 22983114

[JCS259324C11] Guo, H., Chitiprolu, M., Roncevic, L., Javalet, C., Hemming, F. J., Trung, M. T., Meng, L., Latreille, E., Tanese de Souza, C., McCulloch, D. et al. (2017). Atg5 disassociates the V1V0-ATPase to promote exosome production and tumor metastasis independent of canonical macroautophagy. *Dev. Cell* 43, 716-730.e7. 10.1016/j.devcel.2017.11.018 29257951

[JCS259324C12] Haglund, K., Sigismund, S., Polo, S., Szymkiewicz, I., Di Fiore, P. P. and Dikic, I. (2003). Multiple monoubiquitination of RTKs is sufficient for their endocytosis and degradation. *Nat. Cell Biol.* 5, 461-466. 10.1038/ncb983 12717448

[JCS259324C13] Hofmann, K., Tomiuk, S., Wolff, G. and Stoffel, W. (2000). Cloning and characterization of the mammalian brain-specific, Mg2+-dependent neutral sphingomyelinase. *Proc. Natl. Acad. Sci. USA* 97, 5895-5900. 10.1073/pnas.97.11.5895 10823942 PMC18530

[JCS259324C14] Hu, H.-Y., Yu, C.-H., Zhang, H.-H., Zhang, S.-Z., Yu, W.-Y., Yang, Y. and Chen, Q. (2019). Exosomal miR-1229 derived from colorectal cancer cells promotes angiogenesis by targeting HIPK2. *Int. J. Biol. Macromol.* 132, 470-477. 10.1016/j.ijbiomac.2019.03.221 30936013

[JCS259324C15] Hullin-Matsuda, F., Taguchi, T., Greimel, P. and Kobayashi, T. (2014). Lipid compartmentalization in the endosome system. *Semin. Cell Dev. Biol.* 31, 48-56. 10.1016/j.semcdb.2014.04.010 24747366

[JCS259324C16] Huttlin, E. L., Bruckner, R. J., Paulo, J. A., Cannon, J. R., Ting, L., Baltier, K., Colby, G., Gebreab, F., Gygi, M. P., Parzen, H. et al. (2017). Architecture of the human interactome defines protein communities and disease networks. *Nature* 545, 505-509. 10.1038/nature22366 28514442 PMC5531611

[JCS259324C17] Itzhak, D. N., Tyanova, S., Cox, J. and Borner, G. H. H. (2016). Global, quantitative and dynamic mapping of protein subcellular localization. *Elife* 5, 1-36. 10.7554/eLife.16950 PMC4959882 27278775

[JCS259324C18] Kajimoto, T., Okada, T., Miya, S., Zhang, L. and Nakamura, S. I. (2013). Ongoing activation of sphingosine 1-phosphate receptors mediates maturation of exosomal multivesicular endosomes. *Nat. Commun.* 4, 2712. 10.1038/ncomms3712 24231649

[JCS259324C19] Kanada, M., Bachmann, M. H. and Contag, C. H. (2016). Signaling by extracellular vesicles advances cancer hallmarks. *Trends Cancer* 2, 84-94. 10.1016/j.trecan.2015.12.005 28741553

[JCS259324C20] Lafourcade, C., Sobo, K., Kieffer-Jaquinod, S., Garin, J. and van der Goot, F. G. (2008). Regulation of the V-ATPase along the endocytic pathway occurs through reversible subunit association and membrane localization. *PLoS ONE* 3, e2758. 10.1371/journal.pone.0002758 18648502 PMC2447177

[JCS259324C21] Latifkar, A., Ling, L., Hingorani, A., Johansen, E., Clement, A., Zhang, X., Hartman, J., Fischbach, C., Lin, H., Cerione, R. A. et al. (2019). Loss of sirtuin 1 alters the secretome of breast cancer cells by impairing lysosomal integrity. *Dev. Cell* 49, 393-408.e7. 10.1016/j.devcel.2019.03.011 30982660 PMC6519475

[JCS259324C22] Leidal, A. M., Huang, H. H., Marsh, T., Solvik, T., Zhang, D., Ye, J., Kai, F. B., Goldsmith, J., Liu, J. Y., Huang, Y. H. et al. (2020). The LC3-conjugation machinery specifies the loading of RNA-binding proteins into extracellular vesicles. *Nat. Cell Biol.* 22, 187-199. 10.1038/s41556-019-0450-y 31932738 PMC7007875

[JCS259324C23] Levine, B. (2007). Cell biology: autophagy and cancer. *Nature* 446, 745-747. 10.1038/446745a 17429391

[JCS259324C24] Li, X. Q., Liu, J. T., Fan, L. L., Liu, Y., Cheng, L., Wang, F., Yu, H. Q., Gao, J., Wei, W., Wang, H. et al. (2016). Exosomes derived from gefitinib-treated EGFR-mutant lung cancer cells alter cisplatin sensitivity via up-regulating autophagy. *Oncotarget* 7, 24585-24595. 10.18632/oncotarget.8358 27029054 PMC5029725

[JCS259324C25] Lu, F., Liang, Q., Abi-Mosleh, L., Das, A., de Brabander, J. K., Goldstein, J. L. and Brown, M. S. (2015). Identification of NPC1 as the target of U18666A, an inhibitor of lysosomal cholesterol export and Ebola infection. *Elife* 4, 1-16. 10.7554/eLife.12177 PMC4718804 26646182

[JCS259324C26] Matsui, T., Osaki, F., Hiragi, S., Sakamaki, Y. and Fukuda, M. (2021). ALIX and ceramide differentially control polarized small extracellular vesicle release from epithelial cells. *EMBO Rep.* 22, e51475. 10.15252/embr.202051475 33724661 PMC8097368

[JCS259324C27] Menck, K., Sönmezer, C., Worst, T. S., Schulz, M., Dihazi, G. H., Streit, F., Erdmann, G., Kling, S., Boutros, M., Binder, C. et al. (2017). Neutral sphingomyelinases control extracellular vesicles budding from the plasma membrane. *J. Extracell Vesicles* 6, 1378056. 10.1080/20013078.2017.1378056 29184623 PMC5699186

[JCS259324C28] Möbius, W., van Donselaar, E., Ohno-Iwashita, Y., Shimada, Y., Heijnen, H. F. G., Slot, J. W. and Geuze, H. J. (2003). Recycling compartments and the internal vesicles of multivesicular bodies harbor most of the cholesterol found in the endocytic pathway. *Traffic* 4, 222-231. 10.1034/j.1600-0854.2003.00072.x 12694561

[JCS259324C29] Ostrowski, M., Carmo, N. B., Krumeich, S., Fanget, I., Raposo, G., Savina, A., Moita, C. F., Schauer, K., Hume, A. N., Freitas, R. P. et al. (2010). Rab27a and Rab27b control different steps of the exosome secretion pathway. *Nat. Cell Biol.* 12, 19-30. 10.1038/ncb2000 19966785

[JCS259324C30] Panigrahi, G. K., Praharaj, P. P., Peak, T. C., Long, J., Singh, R., Rhim, J. S., Elmageed, Z. Y. A. and Deep, G. (2018). Hypoxia-induced exosome secretion promotes survival of African-American and Caucasian prostate cancer cells. *Sci. Rep.* 8, 1-13. 10.1038/s41598-018-22068-4 29497081 PMC5832762

[JCS259324C31] Parashuraman, S. and D'Angelo, G. (2019). Visualizing sphingolipid biosynthesis in cells. *Chem. Phys. Lipids.* 218, 103-111. 10.1016/j.chemphyslip.2018.11.003 30476485

[JCS259324C32] Philipp, S., Puchert, M., Adam-Klages, S., Tchikov, V., Winoto-Morbach, S., Mathieu, S., Deerberg, A., Kolker, L., Marchesini, N., Kabelitz, D. et al. (2010). The Polycomb group protein EED couples TNF receptor 1 to neutral sphingomyelinase. *Proc. Natl. Acad. Sci. USA* 107, 1112-1117. 10.1073/pnas.0908486107 20080539 PMC2824292

[JCS259324C33] Piper, R. C. and Katzmann, D. J. (2010). Biogenesis and function of MVBs. *Annu. Rev. Cell Dev. Biol.* 23, 519-547. 10.1146/annurev.cellbio.23.090506.123319 PMC2911632 17506697

[JCS259324C34] Raiborg, C. and Stenmark, H. (2009). The ESCRT machinery in endosomal sorting of ubiquitylated membrane proteins. *Nature* 458, 445-452. 10.1038/nature07961 19325624

[JCS259324C35] Robinson, M. W., Alvarado, R., To, J., Hutchinson, A. T., Dowdell, S. N., Lund, M., Turnbull, L., Whitchurch, C. B., O'Brien, B. A., Dalton, J. P. et al. (2012). A helminth cathelicidin-like protein suppresses antigen processing and presentation in macrophages via inhibition of lysosomal vATPase. *FASEB J.* 26, 4614-4627. 10.1096/fj.12-213876 22872675

[JCS259324C36] Roxrud, I., Raiborg, C., Pedersen, N. M., Stang, E. and Stenmark, H. (2008). An endosomally localized isoform of Eps15 interacts with Hrs to mediate degradation of epidermal growth factor receptor. *J. Cell Biol.* 180, 1205-1218. 10.1083/jcb.200708115 18362181 PMC2373575

[JCS259324C37] Saksena, S., Sun, J., Chu, T. and Emr, S. D. (2007). ESCRTing proteins in the endocytic pathway. *Trends Biochem. Sci.* 32, 561-573. 10.1016/j.tibs.2007.09.010 17988873

[JCS259324C38] Sautin, Y. Y., Lu, M., Gaugler, A., Zhang, L. and Gluck, S. L. (2005). Phosphatidylinositol 3-kinase-mediated effects of glucose on vacuolar H+-ATPase assembly, translocation, and acidification of intracellular compartments in renal epithelial cells. *Mol. Cell. Biol.* 25, 575-589. 10.1128/MCB.25.2.575-589.2005 15632060 PMC543406

[JCS259324C39] Sezgin, E., Levental, I., Mayor, S. and Eggeling, C. (2017). The mystery of membrane organization: Composition, regulation and roles of lipid rafts. *Nat. Rev. Mol. Cell Biol.* 18, 361-374. 10.1038/nrm.2017.16 28356571 PMC5500228

[JCS259324C40] Shamseddine, A. A., Airola, M. V. and Hannun, Y. A. (2015). Roles and regulation of neutral sphingomyelinase-2 in cellular and pathological processes. *Adv. Biol. Regul.* 57, 24-41. 10.1016/j.jbior.2014.10.002 25465297 PMC4684640

[JCS259324C41] Taelman, V. F., Dobrowolski, R., Plouhinec, J. L., Fuentealba, L. C., Vorwald, P. P., Gumper, I., Sabatini, D. D. and De Robertis, E. M. (2010). Wnt signaling requires sequestration of Glycogen Synthase Kinase 3 inside multivesicular endosomes. *Cell* 143, 1136-1148. 10.1016/j.cell.2010.11.034 21183076 PMC3022472

[JCS259324C42] Théry, C., Witwer, K. W., Aikawa, E., Alcaraz, M. J., Anderson, J. D., Andriantsitohaina, R., Antoniou, A., Arab, T., Archer, F., Atkin-Smith, G. K. et al. (2018). Minimal information for studies of extracellular vesicles 2018 (MISEV2018): a position statement of the International Society for Extracellular Vesicles and update of the MISEV2014 guidelines. *J. Extracell Vesicles* 7, 1535750. 10.1080/20013078.2018.1535750 30637094 PMC6322352

[JCS259324C43] Toei, M., Saum, R. and Forgac, M. (2010). Regulation and isoform function of the V-ATPases. *Biochemistry* 49, 4715-4723. 10.1021/bi100397s 20450191 PMC2907102

[JCS259324C44] Trajkovic, K. (2008). Ceramide triggers budding of exosome vesicles into multivesicular endosomes (Science (1244)). *Science (80-)* 320, 179. 10.1126/science.320.5873.179 18309083

[JCS259324C45] Trajkovic, K., Hsu, C., Chiantia, S., Rajendran, L., Wenzel, D., Wieland, F., Schwille, P., Brügger, B. and Simons, M. (2008). Ceramide triggers budding of exosome vesicles into multivesicular endosomes. *Science (80-)* 319, 1244-1247. 10.1126/science.1153124 18309083

[JCS259324C46] van Niel, G., D'Angelo, G. and Raposo, G. (2018). Shedding light on the cell biology of extracellular vesicles. *Nat. Rev. Mol. Cell Biol.* 19, 213-228. 10.1038/nrm.2017.125 29339798

[JCS259324C47] Vielhaber, G., Brade, L., Lindner, B., Pfeiffer, S., Wepf, R., Hintze, U., Wittern, K. P. and Brade, H. (2001). Mouse anti-ceramide antiserum: A specific tool for the detection of endogenous ceramide. *Glycobiology* 11, 451-457. 10.1093/glycob/11.6.451 11445550

[JCS259324C48] Villarroya-Beltri, C., Baixauli, F., Mittelbrunn, M., Fernández-Delgado, I., Torralba, D., Moreno-Gonzalo, O., Baldanta, S., Enrich, C., Guerra, S. and Sánchez-Madrid, F. (2016). ISGylation controls exosome secretion by promoting lysosomal degradation of MVB proteins. *Nat. Commun.* 7, 13588. 10.1038/ncomms13588 27882925 PMC5123068

[JCS259324C49] Wang, L., Wu, D., Robinson, C. V., Wu, H. and Fu, T.-M. (2020). Structures of a complete human V-ATPase reveal mechanisms of its assembly. *Mol. Cell* 80, 501-511.e3. 10.1016/j.molcel.2020.09.029 33065002 PMC7655608

[JCS259324C50] Wang, M. X., Cheng, X. Y., Jin, M., Cao, Y. L., Yang, Y. P., Da Wang, J., Li, Q., Wang, F., Hu, L. F. and Liu, C. F. (2015). TNF compromises lysosome acidification and reduces α-synuclein degradation via autophagy in dopaminergic cells. *Exp. Neurol.* 271: 112-121. 10.1016/j.expneurol.2015.05.008 26001614

[JCS259324C51] Wang, R., Wang, J., Hassan, A., Lee, C. H., Xie, X. S. and Li, X. (2021). Molecular basis of V-ATPase inhibition by bafilomycin A1. *Nat. Commun.* 12, 1782. 10.1038/s41467-021-22111-5 33741963 PMC7979754

[JCS259324C52] Werneburg, N. W., Guicciardi, M. E., Bronk, S. F. and Gores, G. J. (2002). Tumor necrosis factor-α-associated lysosomal permeabilization is cathepsin B dependent. *Am. J. Physiol. Gastrointest. Liver Physiol.* 283, 947-956. 10.1152/ajpgi.00151.2002 12223355

[JCS259324C53] Yabu, T., Shiba, H., Shibasaki, Y., Nakanishi, T., Imamura, S., Touhata, K. and Yamashita, M. (2015). Stress-induced ceramide generation and apoptosis via the phosphorylation and activation of nSMase1 by JNK signaling. *Cell Death Differ.* 22, 258-273. 10.1038/cdd.2014.128 25168245 PMC4291487

[JCS259324C54] Yamashita, Y., Kojima, K., Tsukahara, T., Agawa, H., Yamada, K., Amano, Y., Kurotori, N., Tanaka, N., Sugamura, K. and Takeshita, T. (2008). Ubiquitin-independent binding of Hrs mediates endosomal sorting of the interleukin-2 receptor β-chain. *J. Cell Sci.* 121, 1727-1738. 10.1242/jcs.024455 18445679

