# Supplementary material for: Neutral sphingomyelinase 2 controls exosome secretion by counteracting V-ATPase-mediated endosome acidification
Source: J Cell Sci. 2022 Feb 28;135(5):jcs259324. doi: 10.1242/jcs.259324 (PMC8919340; doi:10.1242/jcs.259324)
Supplement: Supplementary information [file joces-135-259324-s1.pdf]

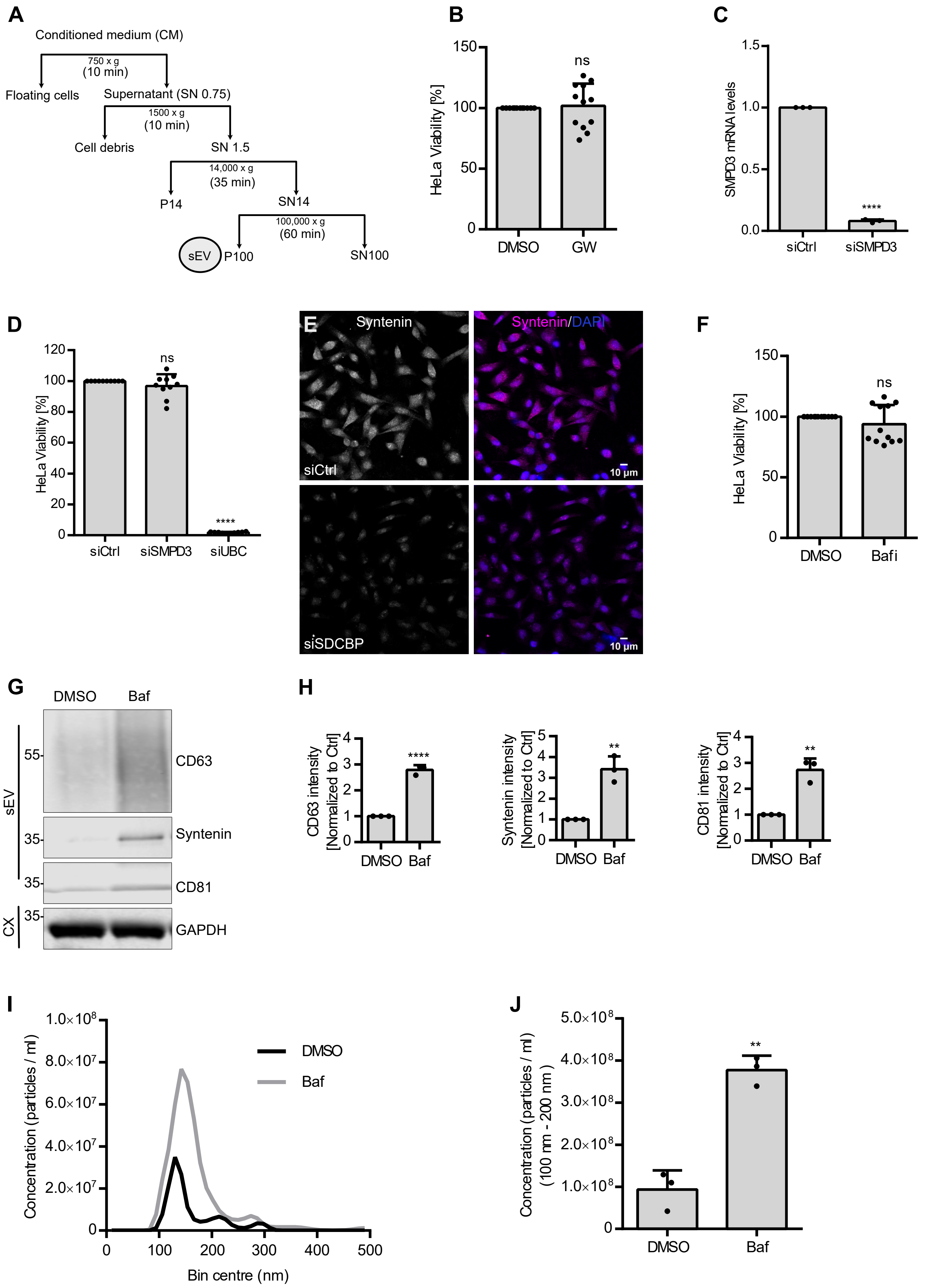

Journal of Cell Science • Supplementary information

Fig. S1. **(A)** Scheme of sEV isolation from cell culture conditioned medium (CM) through stepwise serial centrifugation with duration (min) of each step indicated. The pellet (P) obtained after 100 000 x g centrifugation corresponds to the sEV fraction. **(B)** HeLa cell viability assay upon GW4869 treatment. **(C)** SMPD3 KD efficiency validated by real-time qPCR. **(D)** HeLa cell viability assay upon SMPD3 KD, Ubiquitin KD was used as a negative control. **(E)** Confocal microscopy images of control and syntenin (SDCBP) KD HeLa stained for syntenin and DAPI. **(F)** HeLa cell viability assay upon bafilomycin A1 treatment. **(G)** Western blot analysis of CD63, Syntenin, and CD81 in sEV fractions prepared from equal amounts of DMSO and Bafilomycin A1 (Baf) treated HeLa cells and GAPDH in cell lysate (CX). **(H)** Signal intensity quantifications of CD63, Syntenin, and CD81 in sEV fractions from (G) by normalizing to loading control GAPDH signal in the corresponding cell lysates before normalization to their respective control. **(I)** Representative size distribution of sEV analyzed by NTA. **(J)** NTA quantification of sEV concentration. The data shown (J) represent means ± SD of three biological replicates; ****p < 0,0001, **p < 0,001, *p < 0.01.

Journal of Cell Science • Supplementary information

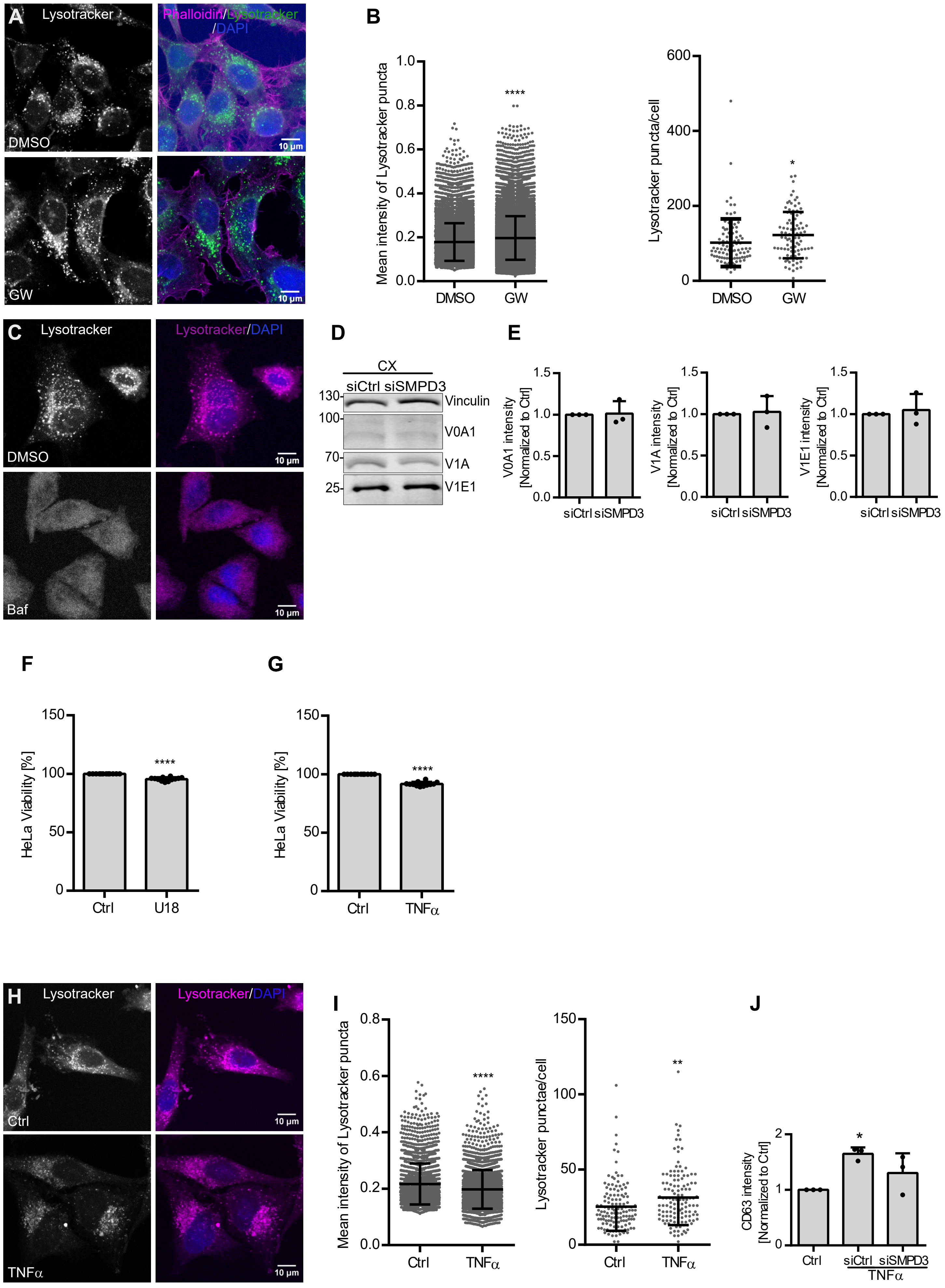

Journal of Cell Science • Supplementary information

Fig. S2. **(A)** Confocal microscopy images of DMSO and GW4869 treated HeLa cells with intracellular acidic compartments labeled by Lysotracker **(B)** Quantifications of mean intensity of Lysotracker punctae (left) and lysotracker staining puncta per cell (right) from (C). (mean ± SD; ****p < 0.0001, Student t-test, n > 50 cells from 3 biological replicates). **(C)** Confocal microscopy images of DMSO and Baf treated HeLa cells with intracellular acidic compartments labeled by Lysotracker. **(D)** Western blot analysis of intracellular V0A1, V1A, and V1E1 in RIPA-cell lysates of HeLa cells. Vinculin was probed as a loading control. **(E)** Quantifications of intracellular V0A1, V1A, and V1E1 in signal intensity from (D) normalized to loading control GAPDH before normalization to the respective control. **(F)** HeLa cell viability assay upon U18 treatment. **(G)** HeLa cell viability assay upon TNFα treatment. **(H)** Confocal microscopy images of control and TNFα treated HeLa cells with intracellular acidic compartments labeled by Lysotracker **(I)** Quantifications of mean intensity of Lysotracker puncta (left) and lysotracker puncta per cell (right) from (H). (mean ± SD; ****p < 0.0001, Student t-test, n > 50 cells from 3 biological replicates). **(J)** Signal intensity quantifications of CD63 in sEV fractions from (Fig. 6G) by normalizing the signal to loading control GAPDH levels in the corresponding cell lysates before normalization to their respective control. The data shown in (E), (F), (G,) and (J) represent means ± SD of three biological replicates; ****p < 0,0001, **p < 0,001, *p < 0.01.
