# Supplementary material for: Neutral sphingomyelinase 2 controls exosome secretion by counteracting V-ATPase-mediated endosome acidification
Source: J Cell Sci. 2022 Feb 28;135(5):jcs259324. doi: 10.1242/jcs.259324 (PMC8919340; doi:10.1242/jcs.259324)
Supplement: Reviewer comments [file joces-135-259324_review_history.pdf]

Neutral sphingomyelinase 2 controls exosome secretion by counteracting V-ATPase-mediated endosome acidification

Dolma Choezom and Julia Christina Gross

DOI: 10.1242/jcs.259324

Editor: James Olzmann

Review timeline

Submission to Review Commons:	17 May 2021
Submission to Journal of Cell Science:	26 August 2021
Editorial decision:	15 November 2021
First revision received:	16 December 2021
Accepted:	29 December 2021

Reviewer 1

Evidence, reproducibility and clarity

Summary

This article by Choezom and Gross aims to understand how nSMase2 affects production of sEVs. They propose that they have identified a mechanism involving control of the V-ATPase activity by budding into intraluminal vesicles of late endosomes.

Major Comments

1. Three inhibitors of nSMase2 are used and a nSMase2 siRNA. With inhibitors, particularly new ones, their specificity is always a concern. Off-target effects can be a concern with siRNA too. Since not all of the inhibitors show the same effects on sEV levels in Fig.1 this suggests that at least some of the inhibitors are acting through a pathway independent of nSMase2. This concern is amplified by the very subtle effects the GW inhibitor has on levels of ceramide in Fig.1I-J. Effects of nSMase2 on levels of sEV markers in Fig.2 are described as "subtle". For many there is no significant difference, despite two of three nSMase2 inhibitors having an effect. These disparities raise serious concerns about the specificity of all results for nSMase2 in Fig.1 and Fig.4.
2. The effects of nSMase2 knockdown on levels of cellular CD63, syntenin etc. is in the order of 10-20%. With these types of subtle differences I am always worried about whether there is some technical or analytical artefact.
3. In experiments with BAF + nSMase2 knockdown one sEV marker is affected (Alix) but not the others. It is very hard to conclude with data like this what the effect is on sEV release, or perhaps whether a subpopulation of sEV release is being affected. Increased numbers of replicates might also make some of the subtle differences in Fig.3C,D become significant and change the interpretation as well.
4. Co-localization of nSMase2 with V0-ATPase A1 subunit, or of ceramide with ATP6AP2 is very challenging to evaluate since there is a lot of signal for both in the nucleus in the images shown. Zoomed in images with co-localization plotted should be provided to show co-localization and the authors should describe how their analysis avoided quantifying the nuclear signal.
5. Much of the microscopy shown does not contain any validation of the specificity of the signaling required. The authors should, for example, knock down antibody targets and show decreased mean intensity of staining in cells. As another example, they should treat with Bafiomycin and show decreased Lysotracker staining.

© 2022. Published by The Company of Biologists under the terms of the Creative Commons Attribution License (https://creativecommons.org/licenses/by/4.0/).

6. The effect on V0A1 levels in sEVs is hard to distinguish in the blot shown, and one almost wonders if the effect is due to poor blotting of the GAPDH in the Control siRNA lane. Moreover, the levels of sEVs are decreasing in nSMase2 knockdown cells, so the effect on V0A1 is likely to be an increase per sEV if normalized to other sEV markers as a loading control. These other markers should be shown in Fig.4D, E.
7. Fig.4G-H. The effects are again subtle and how this is normalized or analyzed would likely change the results and interpretation. If the goal is to analyze what proportion of V-ATPase complexes are intact, the best normalization would probably be to V0A1, but this increases more than V1A and V1E1 in the organelle fractions. This would make it seem like the effect is more on total number of V-ATpase complexes, not on V1 joining to V-ATPase. What is missing in the model is an assessment of whether the amount of V-ATPase internalized by MVB is enough to affect the total organelle levels (lysosome etc.) in Fig.4G-H
8. The effects of the cholesterol inhibitor U18 are also very weak on Lysotracker, organelle associated V-ATPase and sEV release and subject to many of the criticisms above. In addition the effects on sEVs is not unclear as some markers are weakly affected, while others are not affected. The connection between U1186 effects on cholesterol on sphingomyelin and nSMase2 is tenuous.
9. Fig. 6. The authors provide some evidence that TNF increases sEV levels. If nSMase2 is required for sEV release, then one should expect that it will block the increase due to TNF. I'm not convinced that this tells us anything about the mechanism of the TNF effect.
10. In general, the authors use Western blot of multiple markers of sEVs to quantify them. It would have been helpful to have a second method, like nanoparticle tracking to quantify sEVs, particularly when only some of the sEV markers are affected.
11. Lysotracker is not a great indicator of lysosomal pH.
12. Does the author's evidence coalesce around the idea that nSMase2 may affect release of a subset of sEVs, and this may explain why they have effects on only some sEV markers in many experiments?
13. More detail is required on sEV purification protocols. The authors say that cells were grown in 10% FBS, but then say sEVs were purified from EV-free media. What is this media? Time and rotor used for ultracentrifugation also needs to be included at a minimum.
14. More detail is required for how microscopy was quantified. Which pipelines, which settings? Were thresholds or normalization used?
15. Details on drug sources are required.

Significance

This interpretation is debatable and very complicated. For example, not all methods of targeting nSMase2 show the same effects. Many of the effects described by microscopy require more validation of the specificity of reagents and effect sizes are very small. Interpretations of data, particularly around the roles of cholesterol and TNF are very tenuous and there is no great reason to consider them linked to nSMase2 activity. Given these weaknesses it is difficult to assess the contribution to the field.

Referee Cross-commenting

I agree that all 3 reviews raise similar weaknesses in microscopy and quantification of sEV release that put into question the conclusions. To me, the fact that some nSMase2 inhibitors work in some cells, and others work in the other cell type raises serious concerns about which effects are actually due to nSMase2 vs. off-target effects.

Reviewer 2

Evidence, reproducibility and clarity

Summary:

In this work, Choezom and Gross address the mechanisms supporting the effect of nSMase 2 (and also cholesterol) on the secretion of small extracellular vesicles (sEVs). Therefore, they use different drugs known to inhibit the activity of nSMase2 (the long established GW4869, and also

© 2022. Published by The Company of Biologists under the terms of the Creative Commons Attribution License (https://creativecommons.org/licenses/by/4.0/).

more recently identified DPTIP, PDDC), one siRNA smart pool (SMPD3 siRNA), and also TNFa as a way to stimulate nSMase2 activity. They isolated sEVs by differential centrifugation and characterize them by Western Blot using markers enriched in these organelles such as Alix, syntenin, CD63 and sometimes CD81. They also use several confocal microscopy assays.

They propose that nSMase2 acts by supporting the secretion of the vacuolar ATPase integral membrane V0A1 subunit (responsible for H+ translocation) into ILVs, thereby compromising the acidification of multi-vesicular bodies and promoting their fusion with plasma membrane (with as consequence more sEVs secretion) rather than lysosomal degradation.

Experiments are mainly conducted in Hela cells.

The study is interesting and pertinent, yet some claims do not reflect the data presented and some conclusions rely too much on inference.

Major Comments:

In the first part of the study, the authors investigate the effects of 3 different inhibitors of nSMase in 2 different cell types (data shown Fig. 1 and Fig. S1). They conclude that the effects of nSMAse2 inhibition are cell-type specific. This seems indeed to be the case when looking at the impact of GW4869 and DPTIP, but the data show that PDDC has consistent effects on sEV secretion irrespective of the cell type of origin. Moreover, the effects are also different depending on which inhibitor is used. Fig. 1 and S1 are therefore bringing a lot of confusion. It might be more indicated to keep the characterization of different inhibitors for another study?
Of note, the effects of nSMase2 siRNA in Hela are fully consistent with the GW4869 (on cellular and EV extracts).

I think that it is unfortunate that the author did not complement their sEV data with NTA analysis that would directly measure the number of particles. They base all their conclusions on the levels of specific proteins which can be an oversimplification.
It is also unfortunate that the authors used a pool of different siRNA to downregulate nSMase2 rather than studying in parallel the effects of two different siRNA (to discard potential off-target effects).
Fig. 3 C-D is not conclusive. Indeed, one would expect Baf and Baf + siCtrl to give similar results. This is absolutely not the case which makes the interpretation of the authors not acceptable. I would recommend to redo the experiments with lane 1 DMSO-siCtrl, lane 2 DMSO-siSMPD3 rather than what is presented. Here also, the data should ideally be complemented by NTA analysis. Particularly the conclusions are not valid for all markers.

Fig. 3F, please add higher magnification to illustrate colocalization at vesicular cytosolic structures.
Fig. 3G, frankly one wonders whether the colocalization is not mainly coming from the nuclear structures which would completely change the interpretation! Please clarify and/or adjust.

Fig. 4A, images are not directly illustrating the claims, higher magnification might help, same remark as for Fig. 3F-G, make sure nuclear regions are excluded from the statistical analysis.
Fig. 4D-E, frankly the western blot does not illustrate a drop of 50% of the V0A1 signal as shown by the histogram.

The title of Fig. 4 is 'SMPD3 modulates endosomal acidification by V0A1 sequestration into ILV'. The problem is that it is an inference from sEVs marker levels. Please show ILVs by super-resolution and/or electron microscopy, or change claim to statements sticking to the data (and keep inferences/interpretations for the discussion).

Fig. 6A-B Frankly, I don't see the increased colocalization of Ceramide puncta with HRS, yet the decrease of HRS signal upon TNFa is striking.
269 ...increasing V0A1 internalization into ILVs...-> such claim requires direct evidence (super-resolution and/or electron microscopy data), see also above.

© 2022. Published by The Company of Biologists under the terms of the Creative Commons Attribution License (https://creativecommons.org/licenses/by/4.0/).

Minor Comments:

Sometimes authors use Box plots, sometimes not, the rational for that is not clear.

The impact of nSMAse2 silencing on Syntenin, Alix and CD63 levels in sEVs was published in 2012 in Baietti et al., please refer to.

Fig. S2 C-E make clear it is HCT116 data.

Fig. S2G should read S2F.

Fig. S2F is not sufficiently illustrating the claim, add statistics about co-localization.

178 ...'As we found nSMase2 activity to be reduced at HRS-positive endosomes' -> please add reference.

Reference to Fig. 3E.

Fig. 4A add GW on the micrographs.

Fig. 4F partially redundant with Fig. 7, maybe better to have only one summary model.

Fig. 4G-H, 5C-D and 5G-H the Western blots are of poor quality and/or not really consistent with the histograms.

The legend of Fig. S3 is wrong, sometimes ref to Fig. S3 in the text also, remnants of a previous version of the manuscript? Please adjust.

260 Fig 5E, F should be 6 E, F.

Significance

The study is interesting and pertinent for the field of extracellular vesicles, because the molecular mechanisms governing the biology of these organelles are far from completely understood. Yet some claims do not reflect the data presented and some conclusions rely too much on inference. In other words, the work needs to gain in robustness.

I am a cell biologist familiar with the study of the molecular mechanisms governing EV biogenesis, heterogeneity, uptake and signaling. I am familiar with lipid biology.

Referee Cross-commenting

I think the 3 reports are relatively consistent.

Reviewer 3

Evidence, reproducibility and clarity

Summary

In this work, Dr. Choezom and Dr. Gross show that neutral sphingomyelinase 2 (nSMase2), an enzyme which hydrolyzes sphingomyelin in ceramide and phosphorylcholine, plays an important role in the regulation of small EVs secretion in human cancer cell lines. In particular, the authors propose that nSMase2 could drive MVB toward sEV secretion by reducing MVB acidification. The authors propose that this function is associated to the capability of nSMase 2 to keep the V-ATPase transmembrane subunit V0A1 sequestered into Intraluminal Vesicles (ILVs), with less assembled V-ATPase in MVB, less lumen acidification and more EV secretion.

© 2022. Published by The Company of Biologists under the terms of the Creative Commons Attribution License (https://creativecommons.org/licenses/by/4.0/).

Major comments
1) In Figure 1AB and EF and in Supplementary Figure 1BC and FG, the authors show WB analyses of different cytoplasmic or surface EV markers in small EV pellets isolated by ultracentrifugation from conditioned media of colorectal cancer cells (HCT116) or cervical cancer cells (Hela) upon inhibition of nSMase 2 with GW4869 or with specific pharmacological inhibitors DPTIP and PDDC. The authors found that the inhibitors DPTIP and PDDC but not the drug GW4869 are effective on HCT116 derived sEVs for the reduction of the majority of the analyzed markers. Conversely, the authors conclude that the inhibitors DPTIP and PDDC are not very effective in Hela cells whereas GW4869 seemed to be more effective in reducing the expression of the analyzed EV markers. According to MISEV2018 guidelines, the authors should use at least another technique to show that the abovementioned compounds reduce EV secretion. I would strongly suggest the authors to use Nanoparticle Tracking Analysis (NTA) to corroborate that these compounds are effective in reducing EV secretion but also to show which is the average size of the isolated EVs to ensure the quality of their EV preparation. Moreover, the authors should mention from how many secreting cells the WB has been performed. Ideally, in general, a single EV analysis technique as electron microcopy should be presented as a further corroboration of the quality of EV isolation.

2) Could the authors explain why despite DPTIP and PDDC seemed to work in HCT116 derived sEVs they decided to only focus on GW4869 in Hela? Could the authors comment on the different functioning of the inhibitors in different cell lines?

3) In lines 132-133 the authors conclude that: "Collectively, these data suggest that nSMase2 activity at endosomal membranes affects different sEV populations in a cell-type specific manner". I suggest the authors to review these conclusions because a a) WB analysis of some EV markers is not sufficient to conclude that nSMase 2 affects different EV populations without the use of alternative techniques (e.g. EV fractionation or EV co-immunoprecipitation) and b) the specific inhibitors were working somehow also in HCT116 and the authors do not comment on this point in a satisfying manner.

4) As for Figure 1, also the experiments in Figure 2 in which the authors target nsMAse2 with siRNA need a NTA validation to confirm that the inhibition of this molecule leads to reduced EV secretion.

5) Bafilomycin A1 has been shown also recently to induce increase of EV secretion (e.g. Cashikar and Hanson., 2019). In Figure 3C (DMSO vs Bafilomycin) the authors show a convincing increase of CD63, Syntenin and CD81. However, again, the authors should validate the effectiveness of Bafilomycin A1 using NTA besides WB.

6) Figure 4A showing subcellular localization of ATP6AP2 can be confusing, because this ATP6AP2 subunit is not used in further experiments of the figure. Moreover, in all other experiments of the figure the authors analyze the effect of siSMPD3 and only in this one they analyze the effect of GW4869. Could the authors re-evaluate if it is necessary to show this experiment for the flow of the paper?

7) Figure 5 about cholesterol effect: NTA is needed to ascertain that U18 compound is able to affect EV secretion.

8) Increased secretion of EVs upon TNF stimulation or rescue of this effect in siSMPD3 in Figures 6EG and GH, respectively, need to be corroborated by NTA.

Minor comments

1) For Figure 1 and Supplementary Figure 1, I would invite the authors to check more carefully the correspondence between text and figure: in the text, line 111, the authors say that CD81 marker is decreased in HCT116 sEVs blot of Figure 1A and B, however there is no CD81 at all in the referred blot. Moreover, the authors split the HCT116 or Hela blots between Figure 1 and Supplementary Figure 1, making quite complicate for the reader to follow the flow of the experiments, so I would suggest the authors to consider preparing figures in which the different types of inhibitors are side by side compared in the cell lines. Moreover, in Supplementary Figure 1 the authors do not clearly indicate when the blot is from HCT116 and Hela, making further complicate for the reader to understand the differences between the two cell lines.

© 2022. Published by The Company of Biologists under the terms of the Creative Commons Attribution License (https://creativecommons.org/licenses/by/4.0/).

2) In Figure 1I and J the authors propose a quantification of ceramide puncta mean intensity and of colocalization with endosomal protein HRS. Could the authors consider to increase the size of the showed IF images or show a bigger magnification? It is quite hard to see the staining and the relative differences upon GW4869 treatment. Also, the authors should consider to use another type of representation for the quantification rather than the box plot, in which is possible to appreciate more clearly the distribution of the different measured points.

3) For more clarity, I suggest the authors to move the line 138-139 about validation of nsMase2 Si in line 136, before describing the effect of the silencing. Also, could the authors consider to show also a WB of SMPD3 to validate the functioning of the siSMPD3 as done in (Menck et al., 2017)?

4) In Figure 2A and C could the authors specify that the blots are in Hela? As for IF in Figure 1I, also IF in Figures 2EGI are too small and is difficult to appreciate the staining and the differences so I would ask again the authors to at least increase the size of the figure.

5) In Supplementary Figure 2F the authors do not show any quantification of CD63/Lamp1 colocalization in siSMPD3 Hela cells. They should quantify this as done for the other IFs presented in the paper because the extent of colocalization is not very clear in the picture.

6) The resolution and size of lysotracker staining in Fig 3A are not entirely satisfying. Could the authors improve the quality and size of the images? The comment of the way of representing the date is similar to point 2.

7) The Figure 3E is in not mentioned in the text and has no quantification. Please be careful to this.

8) Given that the authors did not author the work (Huttlin et al., 2017) maybe is sufficient to cite it and is not really necessary to put the scheme in the figures of the present paper.

9) Line 195 "could correspond".

10) Figure 5A same comments as for previous similar IF experiments.

11) Line 251 "ccolocalizing".

12) It seems that there is a mistake when authors refer to Fig.6EF about the effect of TNF on EV secretion because the call it Fig 5EF. Please correct.

13) In material and methods section, please indicate the reference of all the antibodies used for WB or IF.

Significance

The group of Dr. Gross already focused on nSMase 2 role in the secretion of EVs budding from plasma membrane (Menk et al., 2017). This time, the results collected in the present work from Dr. Choezom and Dr. Gross highlight the function of the nSMase 2 in the secretion of small EVs of endosomal origin. It was already shown in literature that inhibition of nsMases can reduce the secretion of EVs of endosomal origin (Trajkovic et al.,2008). Here the authors also propose for the first time that this can be correlated to the capability of nSMase 2 to localize the V-ATPase transmembrane subunit V0A1 in ILVs and thus to counteract the function of assembled V-ATPase in MVB, with less acidification and more EV secretion. The obtained results are in line with the current knowledge that reduced acidification of late endosomal compartments could correspond to increased EV secretion, like in the case of the effect induced by Bafilomycin A1 (Cashikar and Hanson., 2019).
Consistently, the authors show that induction of nSMase 2 by TNF increases the secretion of EVs. Overall, the results of this work are quite interesting but some revisions are still needed prior to publication. Once achieved this, this work can be of interest for specialists working in the field of EVs seeking for a tool to modulate EV secretion. I am a PhD working in the field of EVs, with expertise in EV biogenesis and function.

© 2022. Published by The Company of Biologists under the terms of the Creative Commons Attribution License (https://creativecommons.org/licenses/by/4.0/).

Referee Cross-commenting

I particularly agree with reviewer's 2 comments, but also with the majority of reviewer's 1 comments. Overall, this work requires additional effort in quantification of sEV secretion, improvement of confocal microscopy e more attention in negative controls for some of the shown WBs.

Author response to reviewers' comments

Dear Reviewers,

Thank you very much for your detailed feedback on our manuscript.
Please find our point-by-point reply to your comments on the next pages. For clarity, we color-coded the responses: Our detailed responses are written in blue underneath the particular reviewer comment. Comments that require experimental work and how we accomplish/accomplished this are replied in red. Comments that require text editing are replied in green. We edited the manuscript accordingly and highlighted changes in yellow.

Reviewer #1 (Evidence, reproducibility, and clarity (Required)):
Summary
This article by Choezom and Gross aims to understand how nSMase2 affects the production of sEVs. They propose that they have identified a mechanism involving control of the V-ATPase activity by budding into intraluminal vesicles of late endosomes.

Major Comments
1. Three inhibitors of NSMase2 are used and a nSMase2 siRNA. With inhibitors, particularly new ones, their specificity is always a concern. Off-target effects can be a concern with siRNA too. Since not all of the inhibitors show the same effects on sEV levels in Fig.1 this suggests that at least some of the inhibitors are acting through a pathway independent of nSMase2. This concern is amplified by the very subtle effects the GW inhibitor has on levels of ceramide in Fig.1I-J. Effects of nSMase2 on levels of sEV markers in Fig.2 are described as "subtle". For many there is no significant difference, despite two of three nSMase2 inhibitors having an effect. These disparities raise serious concerns about the specificity of all results for nSMase2 in Fig.1 and Fig.4.

Authors: We thank the reviewer for the detailed comments. We agree with the reviewer on some points but respectively disagree with some concerns that are raised above by the reviewer. And would like to address them point by point below.

- Cell type-specific effects of nSMase2 inhibition are known to affect either P100 or P14 and can also increase the secretion of a specific subpopulation, this is not surprising if one considers the differences in lipid composition of different cell types. We understand that this might seems confusing as Figure 1, but indeed reflects the experiences of EV research and is an unsolved open question.
  →These results are now omitted in the present version of the paper as suggested by reviewer 2, but can be included on the editors' advice.

- Reduction of exosome secretion by GW4869 treatment and SMPD3 KD in different cell lines is already known and published in many studies (Baietti *et al*, 2012; Bebelman *et al*, 2020; Van Niel *et al*, 2018). We validated published studies in HeLa cells in Fig 1. We additionally analyzed the effects of GW4869 and SMPD3 KD in HCT116 which was less significant than in HeLa. This is not surprising because it is already known that nSMase2 regulation of exosome biogenesis seems to be cell-type specific (Panigrahi *et al*, 2018).
- Additionally, we do not agree with the reviewer's comment on the reduction of ceramide levels by GW4869 being "subtle". With quantification of ceramide signal in Fig. 2B and with now improved image quality and increased image size in Fig. 2A, we hope one can appreciate the significant reduction of ceramide signal by GW4869 (Fig 2A, B).

© 2022. Published by The Company of Biologists under the terms of the Creative Commons Attribution License (https://creativecommons.org/licenses/by/4.0/).

- We share the concern raised by the reviewer on the specificity of the two new inhibitors of nSMase2– DPTIP, and PDDC. Even though they did not show a consistent result between HeLa and HCT116 cells on sEV secretion, we initially thought that it would be beneficial for the scientific community studying extracellular vesicles to put out these seemingly confusing results. The effects of these inhibitors on both HeLa and HCT116 are omitted as the second reviewer rightfully recommended.

2. The effects of nSMase2 knockdown on levels of cellular CD63, syntenin etc. is in the order of 10-20%. With these types of subtle differences, I am always worried about whether there is some technical or analytical artefact.

Authors: We respectfully disagree with the above comment. A slight reduction in EV level or accumulation in cell extract alone would not be very meaningful, but if simultaneously EV secretion is inhibited, then we would expect both observations together - EV markers reduced in the extracellular milieu and accumulate inside the cells.

- In alignment with the significant reduction of exosome markers in sEV fraction in HeLa cells (All the tested markers were significantly reduced) (Fig. 1E, F), and reduced sEV secretion validated by NTA (Fig. 1G, H). SMPD3 KD significantly accumulated these markers intracellularly (Except for Alix in WB) (Fig. S1F, G). Additionally, MVB accumulation by SMPD3 KD was further validated by confocal microscopy in Fig. 2C-H. With improved image quality and increased image size, we hope that one can appreciate the intracellular accumulation of all three MVB/exosome markers - CD63, Syntenin, and LBPA by SMPD3 KD.

3. In experiments with BAF + nSMase2 knockdown one sEV marker is affected (Alix) but not the others. It is very hard to conclude with data like this what the effect is on sEV release, or perhaps whether a subpopulation of sEV release is being affected. Increased numbers of replicates might also make some of the subtle differences in Fig. 3C, D become significant and change the interpretation as well.

Authors: We cordially agree with the reviewer's concern about the conclusion of these data. Since SMPD3 KD rescued the increased secretion of only Alix by bafilomycin treatment, the interpretation remains quite inconclusive. Since this experiment does not significantly contribute to the coherence of this study, we omitted this part. Instead, to further prove that SMPD3 KD interferes with the endolysosomal acidification, we analyzed how SMPD3 KD affects lysosomes and the possible redirection of MVBs under this condition towards the lysosomes (Novel Fig. 3C-F).

4. Co-localization of nSMase2 with V0-ATPase A1 subunit, or of ceramide with ATP6AP2 is very challenging to evaluate since there is a lot of signal for both in the nucleus in the images shown. Zoomed-in images with co-localization plotted should be provided to show co-localization and the authors should describe how their analysis avoided quantifying the nuclear signal.

Authors: We agree with the reviewer. The strong nuclear signal from nSMase2 is now excluded by using DAPI as the masking object during the pixel-based colocalization quantification. The images showing co-staining between ATP6AP2 and ceramide have been improved and enlarged for better visualization.

5. Much of the microscopy shown does not contain any validation of the specificity of the signaling required. The authors should, for example, knock down antibody targets and show decreased mean intensity of staining in cells. As another example, they should treat with Bafilomycin and show decreased Lysotracker staining.

Authors: We agree with the reviewer's comment. Lysotracker staining is completely abolished by bafilomycin treatment as shown in Fig. S2G. To confirm antibody staining specificity, reduced syntenin (SDCBP) staining upon its KD is included in Fig. S1H. Additionally, we used antibody previously validated, for example for ATP6V1E1 from this study (Guo *et al*, 2017)

© 2022. Published by The Company of Biologists under the terms of the Creative Commons Attribution License (https://creativecommons.org/licenses/by/4.0/).

6. The effect on V0A1 levels in sEVs is hard to distinguish in the blot shown, and one almost wonders if the effect is due to poor blotting of the GAPDH in the Control siRNA lane. Moreover, the levels of sEVs are decreasing in NSmase2 knockdown cells, so the effect on V0A1 is likely to be an increase per sEV if normalized to other sEV markers as a loading control. These other markers should be shown in Fig. 4D, E.

Authors: We understand the reviewer's concern. And we would like to clarify that V0A1 protein levels in the sEV fraction were normalized to the corresponding GAPDH (loading control) in the cell lysate (CX). The authors are fully aware of the poor blotting of control GAPDH the sEV fraction (Fig 4D, E), but the band was not used for quantification. Since V0A1 protein levels in sEV small fraction were reduced by SMPD3 KD, we correspondingly concluded that V0A1 secretion on sEV is nSMase2 dependent. As raised by the other two reviewers as well, we understand that concluding "V0A1 invagination into ILV in a nSmase2-dependent" is rather based on inference. Therefore, we rephrased the text (line 191, 199 and 204) to conclude more carefully, that "nSMase2 regulates sEV secretion by modulating V-ATPase assembly".

7. Fig.4G-H. The effects are again subtle and how this is normalized or analyzed would likely change the results and interpretation. If the goal is to analyze what proportion of V-ATPase complexes are intact, the best normalization would probably be to V0A1, but this increases more than V1A and V1E1 in the organelle fractions. This would make it seem like the effect is more on the total number of V-ATPase complexes, not on V1 joining to V-ATPase. What is missing in the model is an assessment of whether the amount of V-ATPase internalized by MVB is enough to affect the total organelle levels (lysosome etc.) in Fig.4G-H
Authors: We agree with the reviewer, but the interpretation is slightly different V-ATPase complex assembly was stabilized by SMPD3 KD rather than inferring increased recruitment of V1E1. A significant increase of V0A1 in the organelle fraction upon SMPD3 KD indicates increased total V-ATPase complex assembly (Fig. 4H, I).
Therefore, protein levels of the subunits in organelle and cytosolic fractions are normalized to GOSR2 and vinculin respectively.
To further support increased V-ATPase assembly by SMPD3 KD, we now show that V0A1 and V1A colocalization increases upon SMPD3 KD (Fig. 4F, G) by confocal microscopy. As recommended by the reviewer and as expected, SMPD3 KD increased the LAMP1 signal which indicates increased lysosome biogenesis, and a small fraction of accumulated CD63 redirects towards the lysosome under this condition (Fig. 3E-F).

8. The effects of the cholesterol inhibitor U18 are also very weak on Lysotracker, organelle associated V-ATPase and sEV release and subject to many of the criticisms above. In addition, the effects on sEVs is not unclear as some markers are weakly affected, while others are not affected. The connection between U1186 effects on cholesterol on sphingomyelin and NSMase2 is tenuous.
Authors: We respectfully disagree with the reviewer's comment on the effects of U18 on the Lysotracker staining being weak. The quantification showed a significant increase in Lysotracker staining upon U18 treatment. The corresponding images are improved and enlarged for better visualization. But we strongly agree with the reviewer that the effect of exosome marker secretion in the sEV fraction is weak as CD63 and CD81 were not significantly reduced (although U18 significantly reduced Alix and Syntenin secretion).
This claim has now been substantiated by a complementary NTA analysis (Fig. 5G, H).

9. Fig. 6. The authors provide some evidence that TNF increases sEV levels. If nSMase2 is required for sEV release, then one should expect that it will block the increase due to TNF. I'm not convinced that this tells us anything about the mechanism of the TNF effect.

Authors: It was shown previously that TNFα activates nSMase2 (Philipp *et al*, 2010), we further proved this by showing increased ceramide staining upon TNFα (Fig 6A, B).
Furthermore, SMPD3 KD rescued the increased sEV secretion by TNFα (Fig 6G, H), which indicates that the TNFα effect is upstream of nSMase2. We now provide NTA analysis for this experiment as well. Indeed, the increased sEV secretion upon TNFα is confirmed by NTA as recommended by the reviewer and is absence in the absence of nsmase activity (Fig. 6I, J).

© 2022. Published by The Company of Biologists under the terms of the Creative Commons Attribution License (https://creativecommons.org/licenses/by/4.0/).

10. In general, the authors use Western blot of multiple markers of sEVs to quantify them. It would have been helpful to have a second method, like nanoparticle tracking to quantify sEVs, particularly when only some of the sEV markers are affected.

Authors: We agree with the reviewer and all the sEV samples analyzed by immunoblotting in this study will in addition be analyzed by NTA (Nanotracking particle analysis) to quantify both particle number and size distribution.
Please refer to all the novel NTA figures:

- Fig. 1C, D (GW)
- Fig. 1G, H (SMPD3 KD)
- Fig. S1C, D (Bafilomycin)
- Fig. 5G, H (U18)
- Fig. 6I, J (TNFα and SMPD3 KD)

11. Lysotracker is not a great indicator of lysosomal pH.
Authors: We would like to mention that we did not use Lysotracker to measure pH rather used it qualitatively to quantify acidic vesicles.

12. Does the author's evidence coalesce around the idea that nSMase2 may affect release of a subset of sEVs, and this may explain why they have effects on only some sEV markers in many experiments?

Authors: We agree with the reviewer. All the sEV samples analyzed by immunoblotting in this study have now been analyzed by NTA (Nanotracking particle analysis). They are in agreement with individual exosomal markers in the Western Blot analysis. nSMase2 inhibition and SMPD3 KD reduced all the exosome markers tested in HeLa cells. The effect was weak in HCT116 and in breast cancer cells nSMase2 inhibition even increases large EV secretion (Menck *et al*, 2017). We propose that cell-type specific effects of nsmase2 inhibition may arise from the endosomal dynamics and lipid composition of a specific cell type, therefore in some cell- type only a subpopulation of EV may be affected.

13. More detail is required on sEV purification protocols. The authors say that cells were grown in 10% FBS, but then say sEVs were purified from EV-free media. What is this media? Time and rotor used for ultracentrifugation also needs to be included at a minimum.
Authors: We apologize for this omission and now added details about EV-free media in the methods and also added the duration of each centrifugation step in Fig. S1A.

14. More detail is required for how microscopy was quantified. Which pipelines, which settings? Were thresholds or normalization used?
Authors: We apologize for this omission, the details of image quantification will be added with modified pipelines if necessary.

15. Details on drug sources are required.
Authors: We added the text under materials and methods.

Reviewer #1 (Significance (Required)):
This interpretation is debatable and very complicated. For example, not all methods of targeting nSMase2 show the same effects. Many of the effects described by microscopy require more validation of the specificity of reagents and effect sizes are very small. Interpretations of data, particularly around the roles of cholesterol and TNF are very tenuous and there is no great reason to consider them linked to nSMase2.

Referee Cross-commenting
I agree that all 3 reviews raise similar weaknesses in microscopy and quantification of sEV release that put into question the conclusions. To me, the fact that some nSMase2 inhibitors work in some cells, and others work in the other cell type raises serious concerns about which effects are actually due to nSMase2 vs. off-target effects.

© 2022. Published by The Company of Biologists under the terms of the Creative Commons Attribution License (https://creativecommons.org/licenses/by/4.0/).

Reviewer #2 (Evidence, reproducibility and clarity (Required)):

Summary:
In this work, Choezom and Gross address the mechanisms supporting the effect of nSMase 2 (and also cholesterol) on the secretion of small extracellular vesicles (sEVs). Therefore, they use different drugs known to inhibit the activity of nSMase2 (the long established GW4869, and also more recently identified DPTIP, PDDC), one siRNA smart pool (SMPD3 siRNA), and also TNFa as a way to stimulate nSMase2 activity. They isolated sEVs by differential centrifugation and characterize them by Western Blot using markers enriched in these organelles such as Alix, syntenin, CD63 and sometimes CD81. They also use several confocal microscopy assays.
They propose that nSMase2 acts by supporting the secretion of the vacuolar ATPase integral membrane V0A1 subunit (responsible for H+ translocation) into ILVs, thereby compromising the acidification of multi-vesicular bodies and promoting their fusion with plasma membrane (with as consequence more sEVs secretion) rather than lysosomal degradation.
Experiments are mainly conducted in Hela cells.
The study is interesting and pertinent, yet some claims do not reflect the data presented and some conclusions rely too much on inference.

Major Comments:
In the first part of the study, the authors investigate the effects of 3 different inhibitors of nSMase in 2 different cell types (data shown Fig. 1 and Fig. S1).
They conclude that the effects of nSMAse2 inhibition are cell-type specific. This seems indeed to be the case when looking at the impact of GW4869 and DPTIP, but the data show that PDDC has consistent effects on sEV secretion irrespective of the cell type of origin. Moreover, the effects are also different depending on which inhibitor is used. Fig. 1 and S1 are therefore bringing a lot of confusion. It might be more indicated to keep the characterization of different inhibitors for another study?
Of note, the effects of nSMase2 siRNA in Hela are fully consistent with the GW4869 (on cellular and EV extracts).
Authors: We thank the reviewer for the detailed comments on the manuscript. We agree with the reviewer that the effects of these inhibitors are confusing, therefore, in the current version of the manuscript we omitted the DPTIP and PDDC data as suggested.

I think that it is unfortunate that the author did not complement their sEV data with NTA analysis that would directly measure the number of particles. They base all their conclusions on the levels of specific proteins which can be an oversimplification.
It is also unfortunate that the authors used a pool of different siRNA to downregulate nSMase2 rather than studying in parallel the effects of two different siRNA (to discard potential off-target effects).
Authors: We fully agree with the reviewer. All the sEV samples analyzed by immunoblotting in this study have now been analyzed by NTA (Nanotracking particle analysis) as well for the revised version of the manuscript. We used the same SMPD3 siRNA pool that was used in the previous lab publication where we validated the functionality of the four pooled siRNA in breast cancer cells (Menck *et al*, 2017). For the revised version we now added NTA analysis of a deconvoluted pool of siRNA 1 & 2 and 3&4, which both independently reduced the number of secreted sEVs (Fig, 1G,H) as did the pool.

Fig. 3 C-D is not conclusive. Indeed, one would expect Baf and Baf + siCtrl to give similar results. This is not the case which makes the interpretation of the authors not acceptable. I would recommend to redo the experiments with lane 1 DMSO-siCtrl, lane 2 DMSO-siSMPD3 rather than what is presented. Here also, the data should ideally be complemented by NTA analysis. Particularly the conclusions are not valid for all markers.

Authors:
We cordially agree with the reviewer's concern about the conclusion of these data. Since SMPD3 KD rescued the increased secretion of only Alix by bafilomycin treatment, the interpretation remains quite inconclusive. Since this experiment does not significantly contribute to the coherence of this study, we omitted this part. Instead, to further prove that SMPD3 KD interferes with the endolysosomal acidification, we analyzed how SMPD3 KD affects lysosomes and the possible redirection of MVBs under this condition towards the lysosomes (Fig. 3C-F) as suggested

© 2022. Published by The Company of Biologists under the terms of the Creative Commons Attribution License (https://creativecommons.org/licenses/by/4.0/).

by reviewer #1.

Fig. 3F, please add higher magnification to illustrate colocalization at vesicular cytosolic structures. Fig. 3G, frankly one wonders whether the colocalization is not mainly coming from the nuclear structures which would completely change the interpretation! Please clarify and/or adjust.
Authors: We thank the reviewer for the comment. The images have been adjusted and enlarged (Fig. 3G). The strong nuclear signal from nSMase2 is now excluded by using DAPI as the masking object during the pixel-based colocalization quantification. To further prove the interaction between nSMase2 and V0A1, the authors are currently conducting a pulldown experiment to add to the revised version.
Fig. 4A, images are not directly illustrating the claims, higher magnification might help, same remark as for Fig. 3F-G, make sure nuclear regions are excluded from the statistical analysis.
Authors: We thank the reviewer for the comment. All the images in the paper are now adjusted and enlarged.

Fig. 4D-E, frankly the western blot does not illustrate a drop of 50% of the V0A1 signal as shown by the histogram.
Authors: We would like to again clarify that the V0A1 levels in the sEV fraction were normalized to the corresponding GAPDH in the cell lysate where GAPDH levels for siSMPD3 are higher than in the control cells. This could explain the only slight decrease of the V0A1 signal in the sEV fraction upon SMPD3.

The title of Fig. 4 is 'SMPD3 modulates endosomal acidification by V0A1 sequestration into ILV'. The problem is that it is an inference from sEVs marker levels. Please show ILVs by super-resolution and/or electron microscopy, or change claim to statements sticking to the data (and keep inferences/interpretations for the discussion).

Authors: As raised by the other two reviewers as well, we understand this concern. We state more carefully that nSMase activity regulates endolysosomal acidification by modulating V-ATPase assembly.

Fig. 6A-B Frankly, I don't see the increased colocalization of Ceramide puncta with HRS, yet the decrease of HRS signal upon TNFa is striking.
Authors: The images are now adjusted and enlarged (Fig. 6A). Since HRS is also an exosomal marker, decreased HRS signal upon TNFα treated aligns with increased secretion of exosome markers in sEV secretion.

269 ...increasing V0A1 internalization into ILVs...-> such claim requires direct evidence (super-resolution and/or electron microscopy data), see also above.

Authors: As raised by the other two reviewers as well, we understand that concluding "V0A1 invagination into ILV in a nSmase2-dependent" is rather based on inference, however, we find V0A1 signals in the EV fraction, thus secreted from control cells, which is reduced upon nSMase2 inhibition. We state this more carfully in the text now (please refer to lines: 188-194).

Minor Comments:
Sometimes authors use Box plots, sometimes not, the rational for that is not clear.
Authors: We used the box plots to represent results with many data points and bar graphs (showing individual data points) are used for depicting results with less data points.

The impact of nSMAse2 silencing on Syntenin, Alix and CD63 levels in sEVs was published in 2012 in Baietti et al., please refer to.
Authors: We added this reference as suggested.

Fig. S2 C-E make clear it is HCT116 data.
Authors: We now omitted the HCT116 data as suggested by reviewer 2.

Fig. S2G should read S2F.
Authors: Has been corrected.

© 2022. Published by The Company of Biologists under the terms of the Creative Commons Attribution License (https://creativecommons.org/licenses/by/4.0/).

Fig. S2F is not sufficiently illustrating the claim, add statistics about co-localization. Authors: S2F is now moved to Fig 3C with quantifications in Fig 3D.

178 ...'As we found nSMase2 activity to be reduced at HRS-positive endosomes' -> please add reference.
Authors: Corrected

Reference to Fig. 3E.
Authors: The figure has been omitted.
Fig. 4A add GW on the micrographs.
Authors: Corrected

Fig. 4F partially redundant with Fig. 7, maybe better to have only one summary model. Authors: We thank the reviewer for the suggestion and Fig 4F is now removed.

Fig. 4G-H, 5C-D and 5G-H the Western blots are of poor quality and/or not really consistent with the histograms.
Authors: We respectively disagree with the reviewers that WBs 4G-H, 5C-D, and 5 G-H are not consistent with the bar graphs.

The legend of Fig. S3 is wrong, sometimes ref to Fig. S3 in the text also, remnants of a previous version of the manuscript? Please adjust.
Authors: Corrected

260 Fig 5E, F should be 6 E, F.
Authors: This has been corrected.

Reviewer #2 (Significance (Required)):
The study is interesting and pertinent for the field of extracellular vesicles, because the molecular mechanisms governing the biology of these organelles are far from completely understood. Yet some claims do not reflect the data presented and some conclusions rely too much on inference. In other words, the work needs to gain in robustness.

I am a cell biologist familiar with the study of the molecular mechanisms governing EV biogenesis, heterogeneity, uptake and signaling. I am familiar with lipid biology.

Referee Cross-commenting
I think the 3 reports are relatively consistent.
Reviewer #3 (Evidence, reproducibility and clarity (Required)):

Summary
In this work, Dr. Choezom and Dr. Gross show that neutral sphingomyelinase 2 (nSMase2), an enzyme which hydrolyzes sphingomyelin in ceramide and phosphorylcholine, plays an important role in the regulation of small EVs secretion in human cancer cell lines. In particular, the authors propose that nSMase2 could drive MVB toward sEV secretion by reducing MVB acidification. The authors propose that this function is associated to the capability of nSMase 2 to keep the V-ATPase transmembrane subunit V0A1 sequestered into Intraluminal Vesicles (ILVs), with less assembled V-ATPase in MVB, less lumen acidification and more EV secretion.

Major comments
1) In Figure 1AB and EF and in Supplementary Figure 1BC and FG, the authors show WB analyses of different cytoplasmic or surface EV markers in small EV pellets isolated by ultracentrifugation from conditioned media of colorectal cancer cells (HCT116) or cervical cancer cells (Hela) upon inhibition of nSMase 2 with GW4869 or with specific pharmacological inhibitors DPTIP and PDDC. The authors found that the inhibitors DPTIP and PDDC but not the drug GW4869 are effective on HCT116 derived sEVs for the reduction of the majority of the analyzed markers. Conversely, the authors conclude that the inhibitors DPTIP and PDDC are not very effective in Hela cells whereas GW4869 seemed to be more effective in reducing the expression of the analyzed EV markers. According to MISEV2018 guidelines, the authors should use at least another technique to show that

© 2022. Published by The Company of Biologists under the terms of the Creative Commons Attribution License (https://creativecommons.org/licenses/by/4.0/).

the abovementioned compounds reduce EV secretion. I would strongly suggest the authors to use Nanoparticle Tracking Analysis (NTA) to corroborate that these compounds are effective in reducing EV secretion but also to show which is the average size of the isolated EVs to ensure the quality of their EV preparation. Moreover, the authors should mention from how many secreting cells the WB has been performed. Ideally, in general, a single EV analysis technique as electron microcopy should be presented as a further corroboration of the quality of EV isolation.

Authors: We thank the reviewer for the comments and fully agree with the reviewer. We have now analyzed all the sEV samples analyzed by immunoblotting also by NTA (Nanotracking particle analysis) to quantify both particle number and size distribution. The additional data is in agreement with our conclusions.

2) Could the authors explain why despite DPTIP and PDDC seemed to work in HCT116 derived sEVs they decided to only focus on GW4869 in Hela? Could the authors comment on the different functioning of the inhibitors in different cell lines?
Authors: Effects of GW4869 or SMPD3 KD on exosome secretion is better characterized in HeLa cells (Bebelman *et al*, 2020) than in HCT116. We could also validate those published results of reduced sEV secretion in HeLa cells (Fig. 1C,D, G, H). Therefore, we used HeLa cells to further investigate the underlying role of nSMase2 in exosome biogenesis and secretion.
Since these two inhibitors- PDDC and DPTIP- did not phenocopy the effects of GW4869 inhibition in HeLa cells, we could only speculate that the mode of action of these drugs might be different in a cell-type-specific manner from GW4869.
Both SMPD3 KD and GW4869 showed only a slight decrease in sEV secretion in HCT116. This corroborates the findings from other studies where they indicated the cell- type specificity of nSMase2 regulation in exosome biogenesis (Panigrahi *et al*, 2018).

3) In lines 132-133 the authors conclude that: "Collectively, these data suggest that nSMase2 activity at endosomal membranes affects different sEV populations in a cell- type specific manner". I suggest the authors to review these conclusions because a a) WB analysis of some EV markers is not sufficient to conclude that nSMase 2 affects different EV populations without the use of alternative techniques (e.g. EV fractionation or EV co-immunoprecipitation) and b) the specific inhibitors were working somehow also in HCT116 and the authors do not comment on this point in a satisfying manner.
Authors: We agree with the reviewer. Figure 1 is now modified and the conclusion from the figures are also now accordingly changed and corroborated with NTA measurements of both particle number and size distribution.

4) As for Figure 1, also the experiments in Figure 2 in which the authors target nsMAse2 with siRNA need an NTA validation to confirm that the inhibition of this molecule leads to reduced EV secretion.
Authors: We agree with the reviewer and have now included the NTA analysis confirming our conclusions.

5) Bafilomycin A1 has been shown also recently to induce an increase in EV secretion (e.g. Cashikar and Hanson., 2019). In Figure 3C (DMSO vs Bafilomycin) the authors show a convincing increase of CD63, Syntenin, and CD81. However, again, the authors should validate the effectiveness of Bafilomycin A1 using NTA besides WB.

Authors: We fully agree with the reviewer and all the sEV samples analyzed by immunoblotting in this study have now been analyzed by NTA (Nanotracking particle analysis) to quantify both particle number and size distribution.

Please refer to all the novel NTA figures:

- Fig. 1C, D (GW)
- Fig. 1G, H (SMPD3 KD)
- Fig. S1C, D (Bafilomycin)
- Fig. 5G, H (U18)
- Fig. 6I, J (TNFα and SMPD3 KD)

© 2022. Published by The Company of Biologists under the terms of the Creative Commons Attribution License (https://creativecommons.org/licenses/by/4.0/).

6) Figure 4A showing subcellular localization of ATP6AP2 can be confusing, because this ATP6AP2 subunit is not used in further experiments of the figure. Moreover, in all other experiments of the figure the authors analyze the effect of siSMPD3 and only in this one they analyze the effect of GW4869. Could the authors re-evaluate if it is necessary to show this experiment for the flow of the paper?

Authors: nSMase2 activity analysis by using ceramide staining was done with GW4869 throughout the paper. Since ATP6AP2 is also a V-ATPase subunit, we found it important to show that nSMase2 activity targets V-ATPase complex-containing membranes.

7) Figure 5 about cholesterol effect: NTA is needed to ascertain that U18 compound is able to affect EV secretion.
Authors: This has been integrated in novel Fig. 5G, H

8) Increased secretion of EVs upon TNF stimulation or rescue of this effect in siSMPD3 in Figures 6EG and GH, respectively, need to be corroborated by NTA.
Authors: We fully agree with the reviewer and all the sEV samples analyzed by immunoblotting in this study will be analyzed by NTA (Nanotracking particle analysis) to quantify both particle number and size distribution.
Please refer to all the novel NTA figures:

- Fig. 1C, D (GW)
- Fig. 1G, H (SMPD3 KD)
- Fig. S1C, D (Bafilomycin)
- Fig. 5G, H (U18)
- Fig. 6I, J (TNFα and SMPD3 KD)

Minor comments
1) For Figure 1 and Supplementary Figure 1, I would invite the authors to check more carefully the correspondence between text and figure: in the text, line 111, the authors say that CD81 marker is decreased in HCT116 sEVs blot of Figure 1A and B, however there is no CD81 at all in the referred blot. Moreover, the authors split the HCT116 or Hela blots between Figure 1 and Supplementary Figure 1, making quite complicate for the reader to follow the flow of the experiments, so I would suggest the authors to consider preparing figures in which the different types of inhibitors are side by side compared in the cell lines. Moreover, in Supplementary Figure 1 the authors do not clearly indicate when the blot is from HCT116 and Hela, making further complicate for the reader to understand the differences between the two cell lines.
Authors: We apologize for this confusion, as suggested by reviewer 2 we now omitted the HCT116 data and other nSMase inhibitors to focus more on the mechanism of nSMase2 modulating endolysosomal acidification.

2) In Figure 1I and J the authors propose a quantification of ceramide puncta mean intensity and of colocalization with endosomal protein HRS. Could the authors consider to increase the size of the showed IF images or show a bigger magnification? It is quite hard to see the staining and the relative differences upon GW4869 treatment. Also, the authors should consider to use another type of representation for the quantification rather than the box plot, in which is possible to appreciate more clearly the distribution of the different measured points.

Authors: We thank the reviewer for the suggestion. All the images are now improved and enlarged in the paper. We used the box plots to represent results with many data points and bar graphs (showing individual data points) are used for depicting results with fewer data points.

3) For more clarity, I suggest the authors move the line 138-139 about validation of nsMase2 Si in line 136, before describing the effect of the silencing. Also, could the authors consider showing a WB of SMPD3 to validate the functioning of the siSMPD3 as done in (Menck et al., 2017)?
Authors: We moved the text about the validation of siSMPD3 KD as per the reviewer’s suggestion.

© 2022. Published by The Company of Biologists under the terms of the Creative Commons Attribution License (https://creativecommons.org/licenses/by/4.0/).

4) In Figure 2A and C could the authors specify that the blots are in Hela? As for IF in Figure 1I, also IF in Figures 2EGI are too small and is difficult to appreciate the staining and the differences so I would ask again the authors to at least increase the size of the figure.
Authors: We changed the manuscript and only integrated the Hela data as suggested by reviewer 2.

5) In Supplementary Figure 2F the authors do not show any quantification of CD63/Lamp1 colocalization in siSMPD3 Hela cells. They should quantify this as done for the other IFs presented in the paper because the extent of colocalization is not very clear in the picture.
Authors: This has been corrected.

6) The resolution and size of lysotracker staining in Fig 3A are not entirely satisfying. Could the authors improve the quality and size of the images? The comment of the way of representing the date is similar to point 2.

Authors: We used the box plots to represent results with many data points and bar graphs (showing individual data points) are used for depicting results with fewer data points. All images are improved and enlarged.

7) The Figure 3E is in not mentioned in the text and has no quantification. Please be careful to this.
Authors: We omitted Fig. 3E.

8) Given that the authors did not author the work (Huttlin et al., 2017) maybe is sufficient to cite it and is not really necessary to put the scheme in the figures of the present paper.
Authors: We agree with the reviewer, the Figure is now omitted.

9) Line 195 "could correspond".
Authors: This has been corrected.

10) Figure 5A same comments as for previous similar IF experiments.
Authors: This has been corrected.

11) Line 251 "ccolocalizing".
Authors: This has been corrected.

12) It seems that there is a mistake when authors refer to Fig.6EF about the effect of TNF on EV secretion because the call it Fig 5EF. Please correct.
Authors: We corrected this.

13) In material and methods section, please indicate the reference of all the antibodies used for WB or IF.
Authors. We corrected the material and methods section.

Reviewer #3 (Significance (Required)):
Significance
The group of Dr. Gross already focused on nSMase 2 role in the secretion of EVs budding from plasma membrane (Menk et al., 2017). This time, the results collected in the present work from Dr. Choezom and Dr. Gross highlight the function of the nSMase 2 in the secretion of small EVs of endosomal origin. It was already shown in literature that inhibition of nsMases can reduce the secretion of EVs of endosomal origin (Trajkovic et al.,2008). Here the authors also propose for the first time that this can be correlated to the capability of nSMase 2 to localize the V-ATPase transmembrane subunit V0A1 in ILVs and thus to counteract the function of assembled V-ATPase in MVB, with less acidification and more EV secretion. The obtained results are in line with the current knowledge that reduced acidification of late endosomal compartments could correspond to increased EV secretion, like in the case of the effect induced by Bafilomycin A1 (Cashikar and Hanson., 2019). Consistently, the authors show that induction of nSMase 2 by TNF increases the secretion of EVs. Overall, the results of this work are quite interesting but some revisions are still needed prior to publication.

© 2022. Published by The Company of Biologists under the terms of the Creative Commons Attribution License (https://creativecommons.org/licenses/by/4.0/).

Once achieved this, this work can be of interest for specialists working in the field of EVs seeking for a tool to modulate EV secretion. I am a PhD working in the field of EVs, with expertise in EV biogenesis and function.

Referee Cross-commenting
I particulary agree with reviewer's 2 comments, but also with the majority of reviewer's 1 comments. Overall, this work requires additional effort in quantification of sEV secretion, improvement of confocal microscopy e more attention in negative controls for some of the shown WBs.

References

Baietti MF, Zhang Z, Mortier E, Melchior A, Degeest G, Geeraerts A, Ivarsson Y, Depoortere F, Coomans C, Vermeiren E, *et al* (2012) Syndecan-syntenin-ALIX regulates the biogenesis of exosomes. *Nat Cell Biol* 14: 677–685

Bebelman MP, Bun P, Huveneers S, van Niel G, Pegtel DM & Verweij FJ (2020) Real- time imaging of multivesicular body-plasma membrane fusion to quantify exosome release from single cells. *Nat Protoc* 15: 102–121

Guo H, Chitiprolu M, Roncevic L, Javalet C, Hemming FJ, Trung MT, Meng L, Latreille E, Tanese de Souza C, McCulloch D, *et al* (2017) Atg5 Disassociates the V1V0- ATPase to Promote Exosome Production and Tumor Metastasis Independent of Canonical Macroautophagy. *Dev Cell* 43: 716-730.e7

Menck K, Sönmezer C, Worst TS, Schulz M, Dihazi GH, Streit F, Erdmann G, Kling S, Boutros M, Binder C, *et al* (2017) Neutral sphingomyelinases control extracellular vesicles budding from the plasma membrane. *J Extracell Vesicles* 6: 1378056

Van Niel G, D'Angelo G & Raposo G (2018) Shedding light on the cell biology of extracellular vesicles. *Nat Rev Mol Cell Biol* 19: 213–228

Panigrahi GK, Praharaj PP, Peak TC, Long J, Singh R, Rhim JS, Elmageed ZYA & Deep G (2018) Hypoxia-induced exosome secretion promotes survival of African- American and Caucasian prostate cancer cells. *Sci Rep* 8: 1–13

Philipp S, Puchert M, Adam-Klages S, Tchikov V, Winoto-Morbach S, Mathieu S, Deerberg A, Kolker L, Marchesini N, Kabelitz D, *et al* (2010) The Polycomb group protein EED couples TNF receptor 1 to neutral sphingomyelinase. *Proc Natl Acad Sci U S A* 107: 1112–1117

Taelman VF, Dobrowolski R, Plouhinec JL, Fuentealba LC, Vorwald PP, Gumper I, Sabatini DD & De Robertis EM (2010) Wnt signaling requires sequestration of Glycogen Synthase Kinase 3 inside multivesicular endosomes. *Cell* 143: 1136– 1148

Original submission

First decision letter

MS ID#: JOCES/2021/259324

MS TITLE: Neutral Sphingomyelinase 2 controls small extracellular vesicle secretion via counteracting vacuolar ATPase-mediated endosome acidification

AUTHORS: Dolma Choezom and Julia Christina Gross
ARTICLE TYPE: Research Article

We have now reached a decision on the above manuscript.

To see the reviewers' reports and a copy of this decision letter, please go to: https://submit-jcs.biologists.org and click on the 'Manuscripts with Decisions' queue in the Author Area. (Corresponding author only has access to reviews.)

As you will see, the reviewers gave favourable reports and indicated that some of their concerns have been addressed in the revised manuscript. However, both reviewers have some remaining

© 2022. Published by The Company of Biologists under the terms of the Creative Commons Attribution License (https://creativecommons.org/licenses/by/4.0/).

concerns that should be addressed. I hope that you will be able to carry these final comments because I would like to be able to accept your paper.
We are aware that you may be experiencing disruption to the normal running of your lab that makes experimental revisions challenging. If it would be helpful, we encourage you to contact us to discuss your revision in greater detail. Please send us a point-by-point response indicating where you are able to address concerns raised (either experimentally or by changes to the text) and where you will not be able to do so within the normal timeframe of a revision. We will then provide further guidance. Please also note that we are happy to extend revision timeframes as necessary.

Please ensure that you clearly highlight all changes made in the revised manuscript. Please avoid using 'Tracked changes' in Word files as these are lost in PDF conversion.

I should be grateful if you would also provide a point-by-point response detailing how you have dealt with the points raised by the reviewers in the 'Response to Reviewers' box. Please attend to all of the reviewers' comments. If you do not agree with any of their criticisms or suggestions please explain clearly why this is so.

<u>Reviewer 1</u>

Advance summary and potential significance to field

In this work, Dr. Choezom and Dr. Gross show that neutral sphingomyelinase 2 (nSMase2), an enzyme which hydrolyzes sphingomyelin in ceramide and phosphorylcholine, plays an important role in the regulation of small EVs secretion in human cancer cell lines. In particular, the authors propose that nSMase2 could drive MVB toward sEV secretion by reducing MVB acidification. The authors propose that this function is associated to the capability of nSMase 2 to keep the V-ATPase transmembrane subunit V0A1 sequestered into Intraluminal Vesicles (ILVs), with less assembled V-ATPase in MVB, less lumen acidification and more EV secretion. The group of Dr. Gross already focused on nSMase 2 role in the secretion of EVs budding from plasma membrane (Menk et al., 2017). This time, the results collected in the present work from Dr. Choezom and Dr. Gross highlight the function of the nSMase 2 in the secretion of small EVs of endosomal origin. It was already shown in literature that inhibition of nsMases can reduce the secretion of EVs of endosomal origin (Trajkovic et al.,2008). Here the authors also propose for the first time that this can be correlated to the capability of nSMase 2 to localize the V-ATPase transmembrane subunit V0A1 in ILVs and thus to counteract the function of assembled V-ATPase in MVB, with less acidification and more EV secretion. The obtained results are in line with the current knowledge that reduced acidification of late endosomal compartments could correspond to increased EV secretion, like in the case of the effect induced by Bafilomycin A1 (Cashikar and Hanson., 2019). Consistently, the authors show that induction of nSMase 2 by TNF increases the secretion of EVs. Overall, the results of this work are quite interesting but some revisions are still needed prior to publication. Once achieved this, this work can be of interest for specialists working in the field of EVs seeking for a tool to modulate EV secretion.

Comments for the author

The work of Dr. Choezem and Dr. Gross has been already revised by three independent reviewers, thus the authors presented a new corrected version of the manuscript according comments from reviewers. The authors effectively improved the manuscript integrating the majority of suggested changes and required experiments. Despite this, this work still presents some issues.
1) It is becoming a requirement, when publishing on EV biology, to exactely indicate the number of secreting cells used in each experiment. The authors should provide this info in M and M section or in Figure legends. Also especially for the drug GW, it would be good to indicate cell viability for each experiment, to prove that the drug is not inducing cell toxicity which could affect EV secretion.
2) Figure 1A and B: If the authors, as explained in figure legend, really quantified EV markers as ratio on GAPDH in the total lysate shown in supplementary Fig. 1B, they should really show the WBs side by side and not in separate figures. Similar comment for Fig. 5E, 6E.

© 2022. Published by The Company of Biologists under the terms of the Creative Commons Attribution License (https://creativecommons.org/licenses/by/4.0/).

2) Figure 2: the way the authors positioned the IF figures and the relative quantifications is confusing. The authors should position the figures in a more linear manner (e.g. the image and the relative quantification to the right) or to change the letters in order to achieve a more linear order.
3)Suppl.Fig 1H is not well positioned in the page, I was not able to find it immediately. Same comment as before.
4)It seems that in Suppl. Fig. 2E "GW" is not indicated when used in IF picture.
5)Line 151: typo mistake "cellular acidic cellular compartments"
6)Line 162: the statment about autophagy needs a quote 7)Fig. 3: the order of EF GH is confusing, see comment above 8)Fig. 4A: It is not clear if the magnification used in DMSO vs GW is really the same, as in GW it seems that the cells have a reduced size. If this is the effect of the treatment, the authors should comment on this.
9)Figure 4H: The enrichment of V1E1 in the organelle fraction upon siSMPD3 is very difficult to see. Moreover, is there an explaination for the general enrichment of V1E1 (both in SiCnt and siSMPD3) in cytosol more than organelle? this appears not consistent with the blot in Fig. 5C.
10)Figure 4D: despite the quantification of WB shows a reduction of V0A1 upon siSMPD3 it is very hard to see it in the chosen WB. Maybe the authors can choose another representative experiment more consistent with the quantification.
10)NTA measurement in Fig. 6J appears weird. siCnt presents two peaks, with the second one corresponding to larger EVs, which was not the case for siCnt in Fig. 1G. Is there a reason for that?
11) Box plots could not be the ideal way of representing IF quantification data, not allowing to see the actual distribution of the measurements. Yet the authors decided to continue using them with numerous data and used bar graphs with evidenced single points for experiments with few data. For box plots, in figure legends it is not specified the number of replicates, just the number of measured cells. The authors should be more precise and say how many times the experiment has been performed and how many cells have been measured each time.

Reviewer 2

Advance summary and potential significance to field

In this work, Choezom and Gross address the mechanisms supporting the effect of nSMase 2 (and also cholesterol) on the secretion of small extracellular vesicles (sEVs). Therefore, they use a drug known to inhibit the activity of nSMase2 (GW4869), various siRNA combinations against SMPD3, and also TNFa as a way to stimulate nSMase2 activity. They isolated sEVs by differential centrifugation and characterize them by Western Blot using markers enriched in these organelles such as Alix, syntenin, CD63 and sometimes CD81. They also quantify particules by NTA and use several confocal microscopy assays.
They propose that nSMase2 acts by supporting the secretion of the vacuolar ATPase integral membrane V0A1 subunit (responsible for H+ translocation) in small EVs, compromising the acidification of multi-vesicular bodies with as consequence more sEVs secretion, rather than lysosomal degradation. Experiments are mainly conducted in Hela cells. The study is interesting and pertinent, and claims are far better justified than in the previous version.

Comments for the author

Several major comments were properly addressed. I stay with 3 concerns.
Fig. 4D does not illustrate that V0A1 is decreased in siSMPD3 compared to siCtrl. The data (just) show that the siSMPD3 increases the GADPH signal in the cell lysate. Thus, normalizing V0A1 levels in the sEV fraction to the corresponding GAPDH in the cell lysate is putting suspicion, not to say discredit on the way the data were analyzed.
Microscopy images presented figure 3G and 6A are not properly illustrating co-localization. Even after expanding digital original material on my computer screen, I honestly can't convince myself there is more to see there than fortuitous, non-significant co-localization. I strongly recommend to select images with higher magnification, selectively in the 'vesicular areas' clearly illustrating co-localization. Those higher magnifications could for example be added as inserts.

© 2022. Published by The Company of Biologists under the terms of the Creative Commons Attribution License (https://creativecommons.org/licenses/by/4.0/).

First revision

Author response to reviewers' comments

Reviewer 1 Advance Summary and Potential Significance to Field:
In this work, Dr. Choezom and Dr. Gross show that neutral sphingomyelinase 2 (nSMase2), an enzyme which hydrolyzes sphingomyelin in ceramide and phosphorylcholine, plays an important role in the regulation of small EVs secretion in human cancer cell lines. In particular, the authors proposethat nSMase2 could drive MVB toward sEV secretion by reducing MVB acidification. The authors propose that this function is associated to the capability of nSMase 2 to keep the V-ATPase transmembrane subunit V0A1 sequestered into Intraluminal Vesicles (ILVs), with less assembled V-ATPase in MVB, less lumen acidification and more EV secretion. The group of Dr.Gross already focused on nSMase 2 role in the secretion of EVs budding from plasma membrane (Menk et al., 2017). This time, the results collected in the present work from Dr. Choezom and Dr. Gross highlight the function of the nSMase 2 in the secretion of small EVs of endosomal origin. It was already shown in literature that inhibition of nsMases can reduce the secretion of EVs of endosomal origin (Trajkovic et al.,2008). Here the authors also propose for the first time that this can be correlated to the capability of nSMase 2 to localize the V-ATPase transmembrane subunit V0A1 in ILVs and thus to counteract the function of assembled V- ATPase in MVB, with less acidification and more EV secretion. The obtained results are in line with the current knowledge that reduced acidification of late endosomal compartments could correspond to increased EV secretion, like in the case of the effect induced by Bafilomycin A1 (Cashikar and Hanson., 2019) Consistently, the authors show that induction of nSMase 2 by TNF increases the secretion of EVs. Overall, the results of this work are quite interesting but some revisions are still needed prior to publication. Once achieved this, this work can be of interest for specialists working in the field of EVs seeking for a tool to modulate EV secretion.

Reviewer 1 Comments for the Author:
The work of Dr. Choezom and Dr. Gross has been already revised by three independent reviewers, thus the authors presented a new corrected version of the manuscript according comments from reviewers. The authors effectively improved the manuscript integrating the majority of suggested changes and required experiments. Despite this, this work still presents some issues.
1) It is becoming a requirement, when publishing on EV biology, to exactly indicate the number of secreting cells used in each experiment. The authors should provide this info in M and M section or in Figure legends. Also, especially for the drug GW, it would be good to indicate cell viability for each experiment, to prove that the drug is not inducing cell toxicity which could affect EV secretion.
We added the information about cell number to the M&M section and now provide additional viability assays in Figures S1B, D, F and S2F, G.

2) Figure 1A and B: If the authors, as explained in figure legend, really quantified EV markers as ratio on GAPDH in the total lysate shown in supplementary Fig. 1B, they should really show the WBs side by side and not in separate figures. Similar comment for Fig. 5E, 6E.
We have rearranged all Figures and now show WB of Cell extracts and EVs together in one figure along with the quantification.

2) Figure 2: the way the authors positioned the IF figures and the relative quantifications is confusing. The authors should position the figures in a more linear manner (e.g. the image and the relative quantification to the right) or to change the letters in order to achieve a more linear order.
We have rearranged all Figures to present data in a more linear manner.

3) Suppl.Fig 1H is not well positioned in the page, I was not able to find it immediately. Same comment as before.
As suggested we have also arranged the Supplementary Figures in a more comprehensive manner.

4) It seems that in Suppl. Fig. 2E "GW" is not indicated when used in IF picture.
This has been corrected in the new version.

© 2022. Published by The Company of Biologists under the terms of the Creative Commons Attribution License (https://creativecommons.org/licenses/by/4.0/).

5) Line 151: typo mistake "cellular acidic cellular compartments"
This has been corrected.

6) Line 162: the statment about autophagy needs a quote
This has been corrected.

7) Fig. 3: the order of EF GH is confusing, see comment above
This has been corrected.

8) Fig. 4A: It is not clear if the magnification used in DMSO vs GW is really the same, as in GW it seems that the cells have a reduced size. If this is the effect of the treatment, the authors should comment on this.
We apologize, this impression might stem from a different confocal plane of the GW-treated cells, we have exchanged the image in Fig. 4A for a similar confocal plane as seen in the control.

9) Figure 4H: The enrichment of V1E1 in the organelle fraction upon siSMPD3 is very difficult to see. Moreover, is there an explanation for the general enrichment of V1E1 (both in SiCtrl and siSMPD3) in cytosol more than organelle? this appears not consistent with the blot in Fig. 5C. We agree with the reviewer that the increase in E1 in the organelle fraction is rather mild. As E1 is present in the cytoplasm as well as on endosomal membranes (Sautin *et al*, 2005; Toei *et al*, 2010; Guo *et al*, 2017), fractionation in both samples is subject to variations. However, we get a similar slight increase on organelle in agreement with immunofluorescence of E1 in puncta upon SMPD3 KD.

10) Figure 4D: despite the quantification of WB shows a reduction of V0A1 upon siSMPD3 it is very hard to see it in the chosen WB. Maybe the authors can choose another representative experiment more consistent with the quantification.
We found a reproducible, mild reduction of V0A1 in EV upon SMPD3 KD, as the reviewer suggested we exchanged the WB.

10) NTA measurement in Fig. 6J appears weird. siCnt presents two peaks, with the second one corresponding to larger EVs, which was not the case for siCnt in Fig. 1G. Is there a reason for that?
The NTA data was obtained from frozen samples on different days, we took care to always measure samples from the same experiment at the same time. Indeed, a slight shift in the peak of siCTRL is seen in Fig. 6J, but it is within the described size range of exosomes (100 – 200 nm).

11) Box plots could not be the ideal way of representing IF quantification data, not allowing to see the actual distribution of the measurements. Yet, the authors decided to continue using them with numerous data and used bar graphs with evidenced single points for experiments with few data. For box plots, in figure legends it is not specified the number of replicates, just the number of measured cells. The authors should be more precise and say how many times the experiment has been performed and how many cells have been measured each time.
We agree with the reviewer and have changed the IF data to dots blots and indicated the number of times the experiments were done in the figure legend along with the number of cells quantified.

Reviewer 2 Advance Summary and Potential Significance to Field:

In this work, Choezom and Gross address the mechanisms supporting the effect of nSMase 2 (and also cholesterol) on the secretion of small extracellular vesicles (sEVs). Therefore, they use a drug known to inhibit the activity of nSMase2 (GW4869), various siRNA combinations against SMPD3, and also TNFa as a way to stimulate nSMase2 activity. They isolated sEVs by differential centrifugation and characterize them by Western Blot using markers enriched in these organelles such as Alix, syntenin, CD63 and sometimes CD81. They also quantify particles by NTA and use several confocal microscopy assays.

They propose that nSMase2 acts by supporting the secretion of the vacuolar ATPase integral membrane V0A1 subunit (responsible for H+ translocation) in small EVs, compromising the acidification of multi-vesicular bodies with as consequence more sEVs secretion, rather than lysosomal degradation. Experiments are mainly conducted in Hela cells. The study is interesting and pertinent, and claims are far better justified than in the previous version.

© 2022. Published by The Company of Biologists under the terms of the Creative Commons Attribution License (https://creativecommons.org/licenses/by/4.0/).

Reviewer 2 Comments for the Author:
Several major comments were properly addressed. I stay with 3 concerns.
Fig. 4D does not illustrate that V0A1 is decreased in siSMPD3 compared to siCtrl. The data (just) show that the siSMPD3 increases the GADPH signal in the cell lysate. Thus, normalizing V0A1 levels in the sEV fraction to the corresponding GAPDH in the cell lysate is putting suspicion, not to say discredit on the way the data were analyzed.
As mentioned above, we found a reproducible, mild reduction of V0A1 in EV upon SMPD3 KD, as the reviewer 1 suggested we exchanged the WB.

Microscopy images presented figure 3G and 6A are not properly illustrating co-localization. Even after expanding digital original material on my computer screen, I honestly can't convince myself there is more to see there than fortuitous, non-significant co-localization. I strongly recommend to select images with higher magnification, selectively in the 'vesicular areas' clearly illustrating co-localization. Those higher magnifications could for example be added as inserts.
We agree with the reviewer and have integrated larger insets in the figures (Fig.3G, 4F, 6A) to emphasize colocalization in the vesicular areas.

References:

Guo H, Chitiprolu M, Roncevic L, Javalet C, Hemming FJ, Trung MT, Meng L, Latreille E, Tanese de Souza C, McCulloch D, *et al* (2017) Atg5 Disassociates the V1V0-ATPase to Promote Exosome Production and Tumor Metastasis Independent of Canonical Macroautophagy. *Dev Cell* 43: 716-730.e7

Sautin YY, Lu M, Gaugler A, Zhang L & Gluck SL (2005) Phosphatidylinositol 3-Kinase- Mediated Effects of Glucose on Vacuolar H+-ATPase Assembly, Translocation, and Acidification of Intracellular Compartments in Renal Epithelial Cells. *Mol Cell Biol* 25: 575– 589

Toei M, Saum R & Forgac M (2010) Regulation and isoform function of the V-ATPases. *Biochemistry* 49: 4715-723

Second decision letter

MS ID#: JOCES/2021/259324

MS TITLE: Neutral Sphingomyelinase 2 controls exosomes secretion via counteracting V- ATPase-mediated endosome acidification

AUTHORS: Dolma Choezom and Julia Christina Gross
ARTICLE TYPE: Research Article

Thank you for sending your manuscript to Journal of Cell Science through Review Commons.

Thank you for submitting this interesting research to the Journal of Cell Science and for your thoughtful responses to the referees' comments. I am happy to tell you that your manuscript has been accepted for publication in Journal of Cell Science, pending standard ethics checks.

© 2022. Published by The Company of Biologists under the terms of the Creative Commons Attribution License (https://creativecommons.org/licenses/by/4.0/).
